# Author Correction: Gastroesophageal reflux GWAS identifies risk loci that also associate with subsequent severe esophageal diseases

**DOI:** 10.1038/s41467-019-13526-2

**Published:** 2019-12-04

**Authors:** Jiyuan An, Puya Gharahkhani, Matthew H. Law, Jue-Sheng Ong, Xikun Han, Catherine M. Olsen, Rachel E. Neale, John Lai, Tom L. Vaughan, Ines Gockel, René Thieme, Anne C. Böhmer, Janusz Jankowski, Rebecca C. Fitzgerald, Johannes Schumacher, Claire Palles, BEACON, Marilie D. Gammon, Douglas A. Corley, Nicholas J. Shaheen, Nigel C. Bird, Laura J. Hardie, Liam J. Murray, Brian J. Reid, Wong-Ho Chow, Harvey A. Risch, Weimin Ye, Geoffrey Liu, Yvonne Romero, Leslie Bernstein, Anna H. Wu, 23andMe Research Team, M. Agee, B. Alipanahi, A. Auton, R. K. Bell, K. Bryc, S. L. Elson, P. Fontanillas, N. A. Furlotte, D. A. Hinds, K. E. Huber, A. Kleinman, N. K. Litterman, M. H. McIntyre, J. L. Mountain, E. S. Noblin, C. A. M. Northover, S. J. Pitts, J. Fah Sathirapongsasuti, O. V. Sazonova, J. F. Shelton, S. Shringarpure, C. Tian, J. Y. Tung, V. Vacic, C. H. Wilson, David C. Whiteman, Stuart MacGregor

**Affiliations:** 1 Statistical Genetics, QIMR Berghofer Medical Research Institute, Brisbane, QLD Australia; 2 Cancer Control, QIMR Berghofer Medical Research Institute, Brisbane, QLD Australia; 3 Cancer Aetiology and Prevention, QIMR Berghofer Medical Research Institute, Brisbane, QLD Australia; 4 School of Public Health, The University of Queensland, Brisbane, QLD Australia; 5 School of Public Health and Social Work, the Queensland University of Technology, Brisbane, QLD Australia; 6 Centre for Epidemiology and Biostatistics, The University of Melbourne, Melbourne, VIC Australia; 7 Division of Public Health Sciences, Fred Hutchinson Cancer Research Center, Seattle, WA USA; 8 Department of Visceral, Transplant, Thoracic and Vascular Surgery, University Hospital of Leipzig, Leipzig, Germany; 9 Institute of Human Genetics, University of Bonn, School of Medicine & University Hospital Bonn, Bonn, Germany; 10 Department of Genomics, Life & Brain Center, University of Bonn, Bonn, Germany; 11 Royal College of Surgeons in Ireland, Dublin, Ireland; 12 Medical Research Council (MRC) Cancer Unit, Hutchison-MRC Research Centre and University of Cambridge, Cambridge, UK; 13 Center for Human Genetics, University Hospital of Marburg, Marburg, Germany; 14 Institute of Cancer and Genomic Sciences, University of Birmingham, Birmingham, UK; 15 International Barrett’s and Esophageal Adenocarcinoma Consortium, Mountain View, CA USA; 16 23andMe, Inc., Mountain View, CA 94041 USA

Correction to: *Nature Communications* 10.1038/s41467-019-11968-2, published online 16 September 2019.

The original version of this Article omitted from the author list the 10th and 11th authors, Ines Gockel and René Thieme, who are from the Department of Visceral, Transplant, Thoracic and Vascular Surgery, University Hospital of Leipzig, Leipzig, Germany. Consequently, their initials were added to the Author Contributions as having ‘contributed to the data collection and contributed to genotyping.’ This has been corrected in both the PDF and HTML versions of the Article.

