# Supplementary material for: Gastroesophageal reflux GWAS identifies risk loci that also associate with subsequent severe esophageal diseases
Source: Nat Commun. 2019 Sep 16;10:4219. doi: 10.1038/s41467-019-11968-2 (PMC6746768; doi:10.1038/s41467-019-11968-2)
Supplement: Supplementary file 1 — Supplementary Information [file 41467_2019_11968_MOESM1_ESM.pdf]

An *et al*. Gastroesophageal reflux GWAS identifies first risk loci that also associate with subsequent severe esophageal diseases

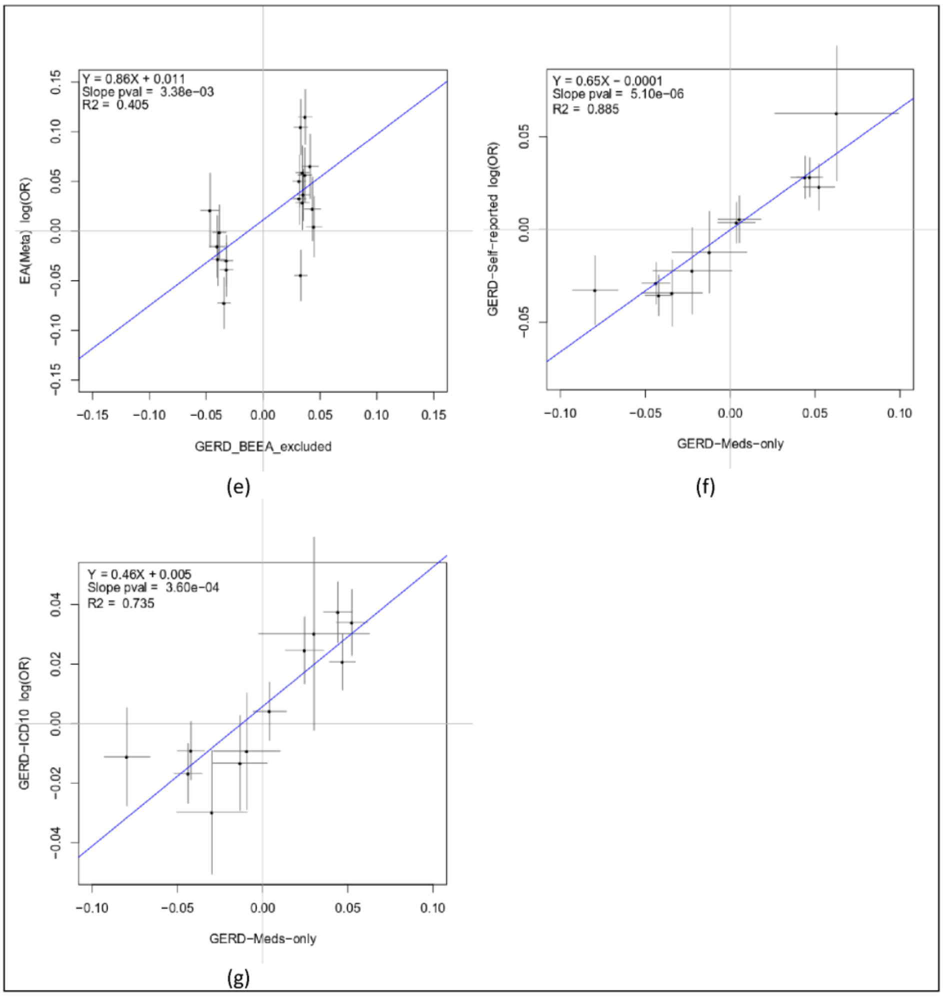

Supplementary Figure 1. (a)The correlation of UKBB GERD vs 23andMe. (b)The correlation of UKBB GERD vs QSKIN GERD. (c) The correlation of Meta GERD (BE/EA samples excluded) vs Meta BEEA. (d) The correlation of Meta GERD (BE/EA samples excluded) vs Meta BE. (e) The correlation of Meta GERD (BE/EA samples excluded) vs EA. Log of significant independent SNPs from GCTA COJO are represented on the x-axis. The corresponding logOR of SNPs from 23andMe and QSKIN are shown on the y-axis. The lengths of the lines for each SNP represent the standard error for the respective logOR. Linear regression was undertaken using the weight of the MAF variance (MAF *(1-MAF)). (f) The correlation of GERD from medicine pheno type only (self-report GERD and ICD10 medical recored samples excluded) vs GERD from self-reported pheno type only. (g) The correlation of GERD from medicine pheno type only (self-report GERD and ICD10 medical recored samples excluded) vs GERD from ICD10 medical record pheno type only.

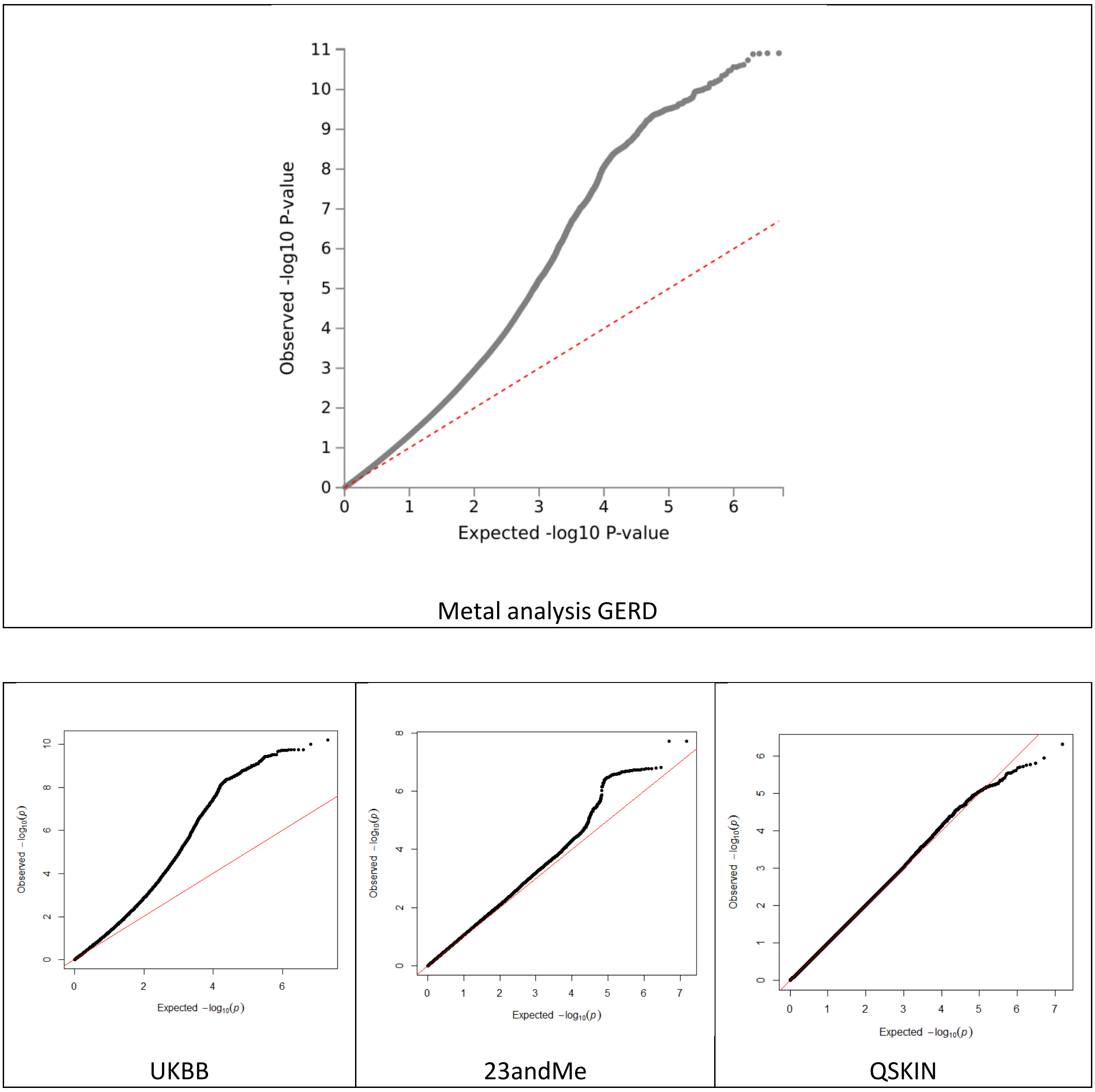

Supplementary Figure 2. QQ plot of GERD GWAS using summary statistics.

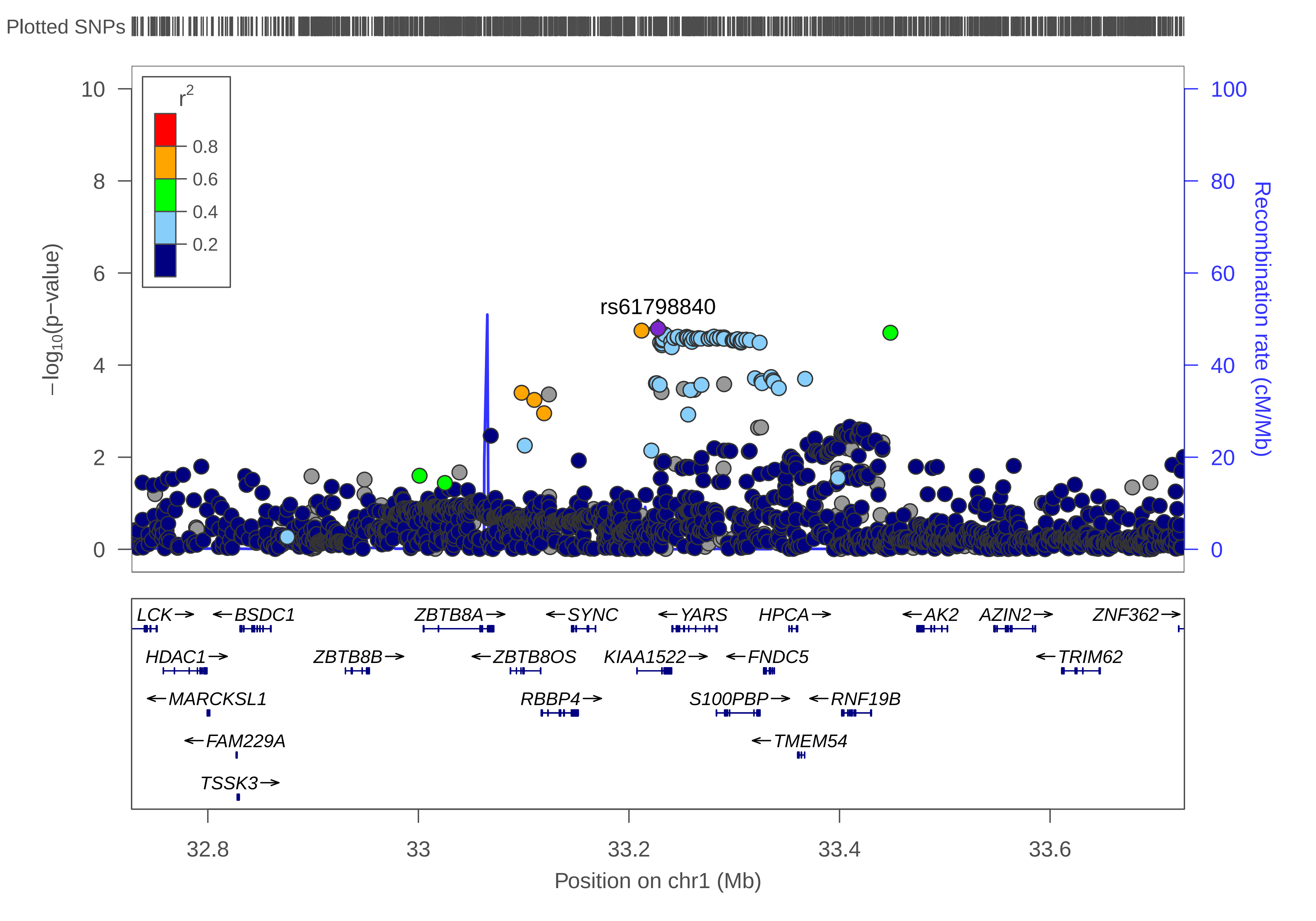

Plotted SNPs
r2
0.8
0.6
0.4
0.2
rs61798840
−log10(p−value)
Recombination rate (cM/Mb)
10
8
6
4
2
0
100
80
60
40
20
0
LCK→
←BSDC1
ZBTB8A→
←SYNC
←YARS
HPCA→
←AK2
AZIN2→
ZNF362→
HDAC1→
ZBTB8B→
←ZBTB8OS
KIAA1522→
←FNDC5
←TRIM62
←MARCKSL1
RBBP4→
S100PBP→
←RNF19B
←FAM229A
←TMEM54
TSSK3→
32.8
33
33.2
33.4
33.6
Position on chr1 (Mb)

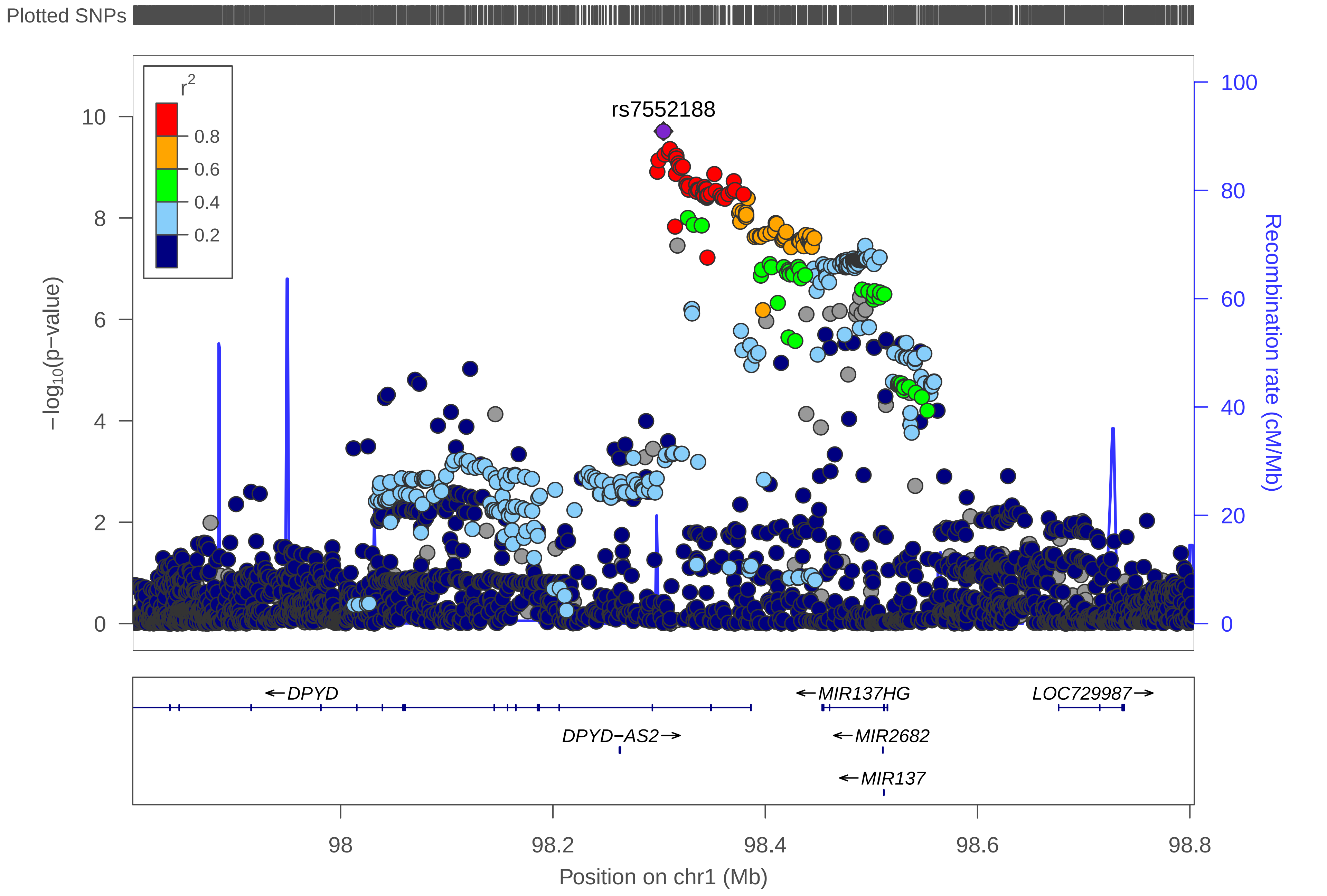

Plotted SNPs
rs7552188
r2
0.8
0.6
0.4
0.2
−log10(p−value)
Recombination rate (cM/Mb)
0
2
4
6
8
10
0
20
40
60
80
100
←DPYD
DPYD−AS2→
←MIR137HG
←MIR2682
←MIR137
LOC729987→
98
98.2
98.4
98.6
98.8
Position on chr1 (Mb)

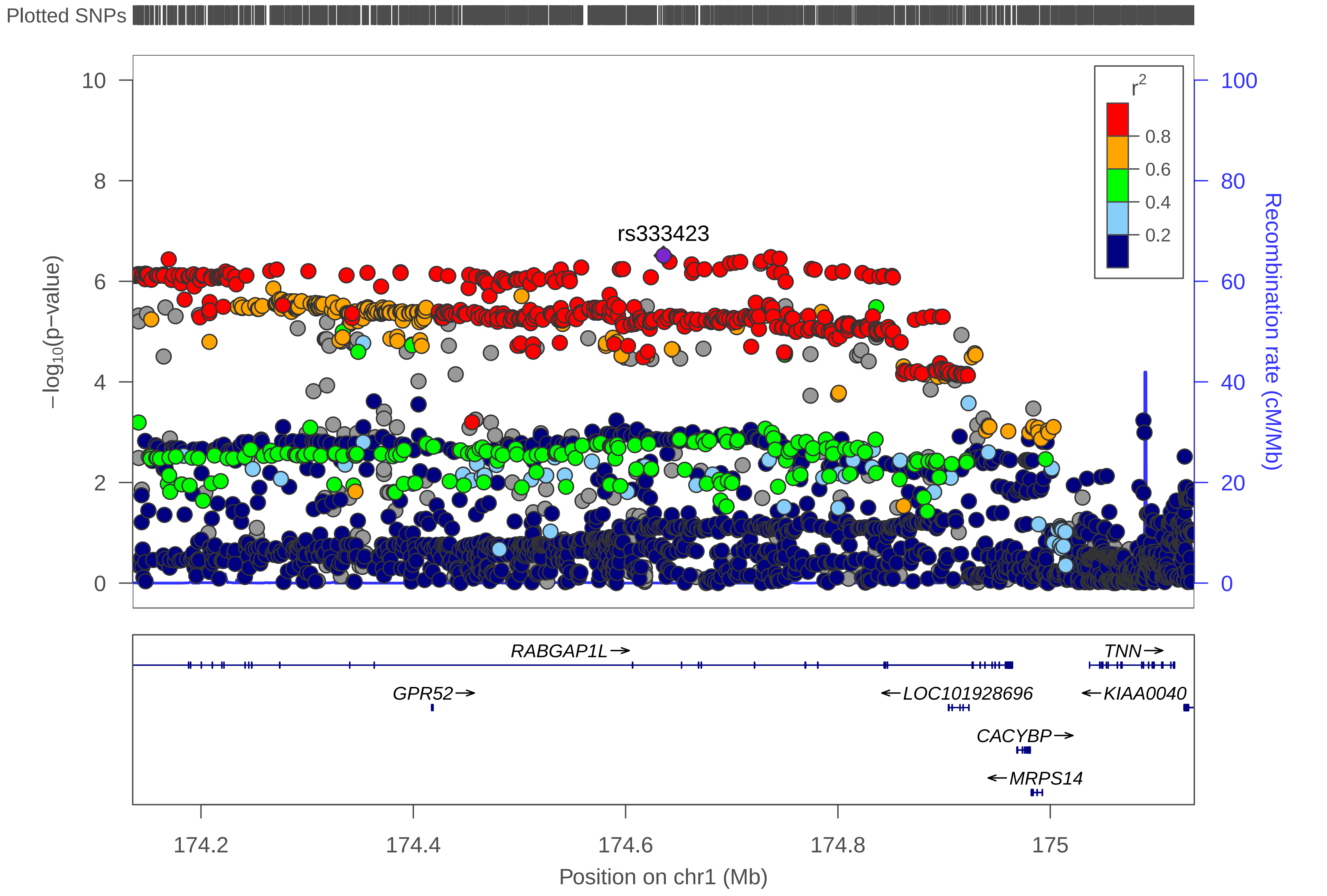

Plotted SNPs
rs333423
r2
0.8
0.6
0.4
0.2
−log10(p−value)
Recombination rate (cM/Mb)
10
8
6
4
2
0
100
80
60
40
20
0
RABGAP1L→
TNN→
GPR52→
←LOC101928696
←KIAA0040
CACYBP→
←MRPS14
174.2
174.4
174.6
174.8
175
Position on chr1 (Mb)

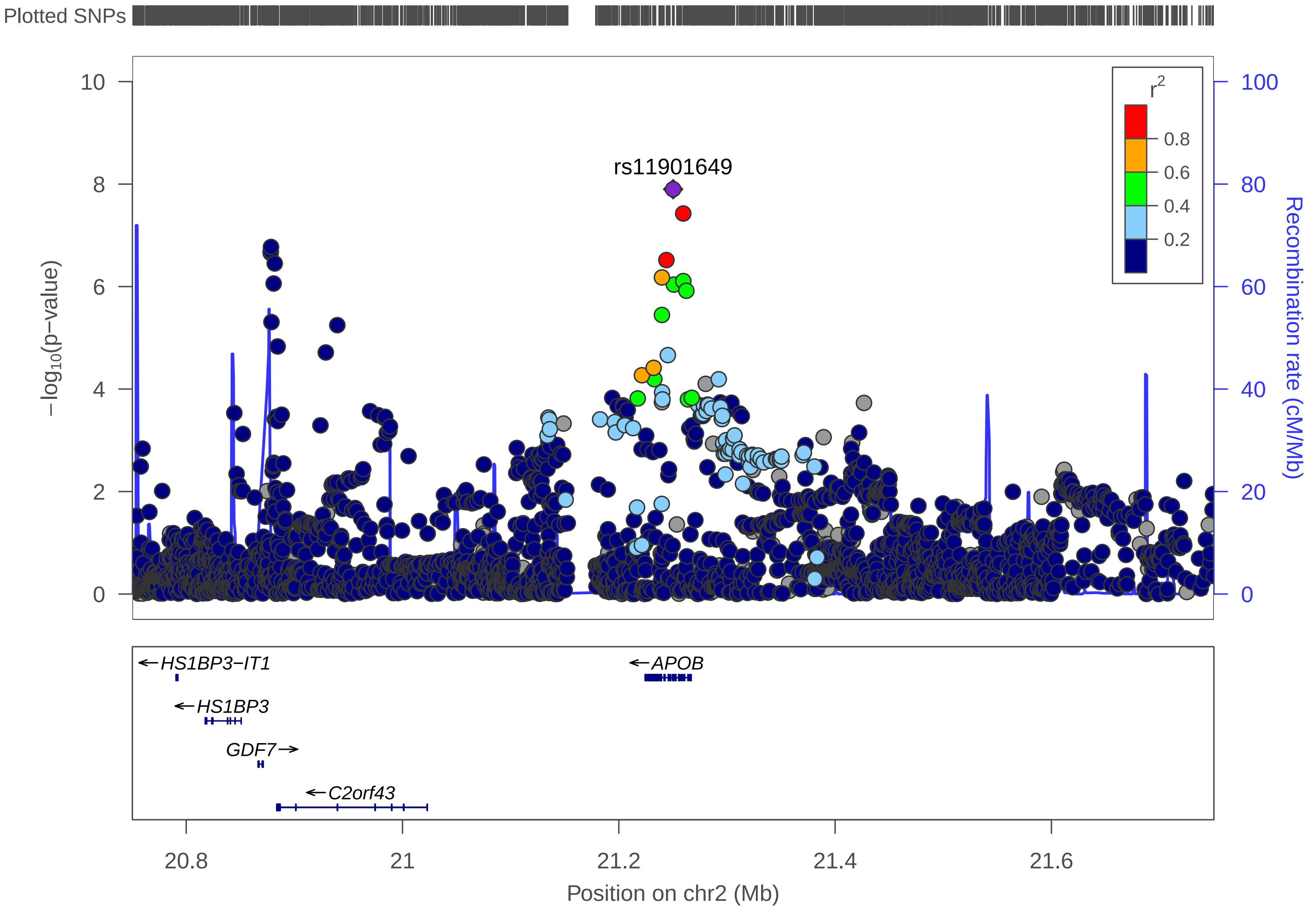
Plotted SNPs
rs11901649
r²
0.8
0.6
0.4
0.2
10
8
6
4
2
0
100
80
60
40
20
0
−log10(p−value)
Recombination rate (cM/Mb)
HS1BP3−IT1
HS1BP3
GDF7
C2orf43
APOB
20.8
21
21.2
21.4
21.6
Position on chr2 (Mb)

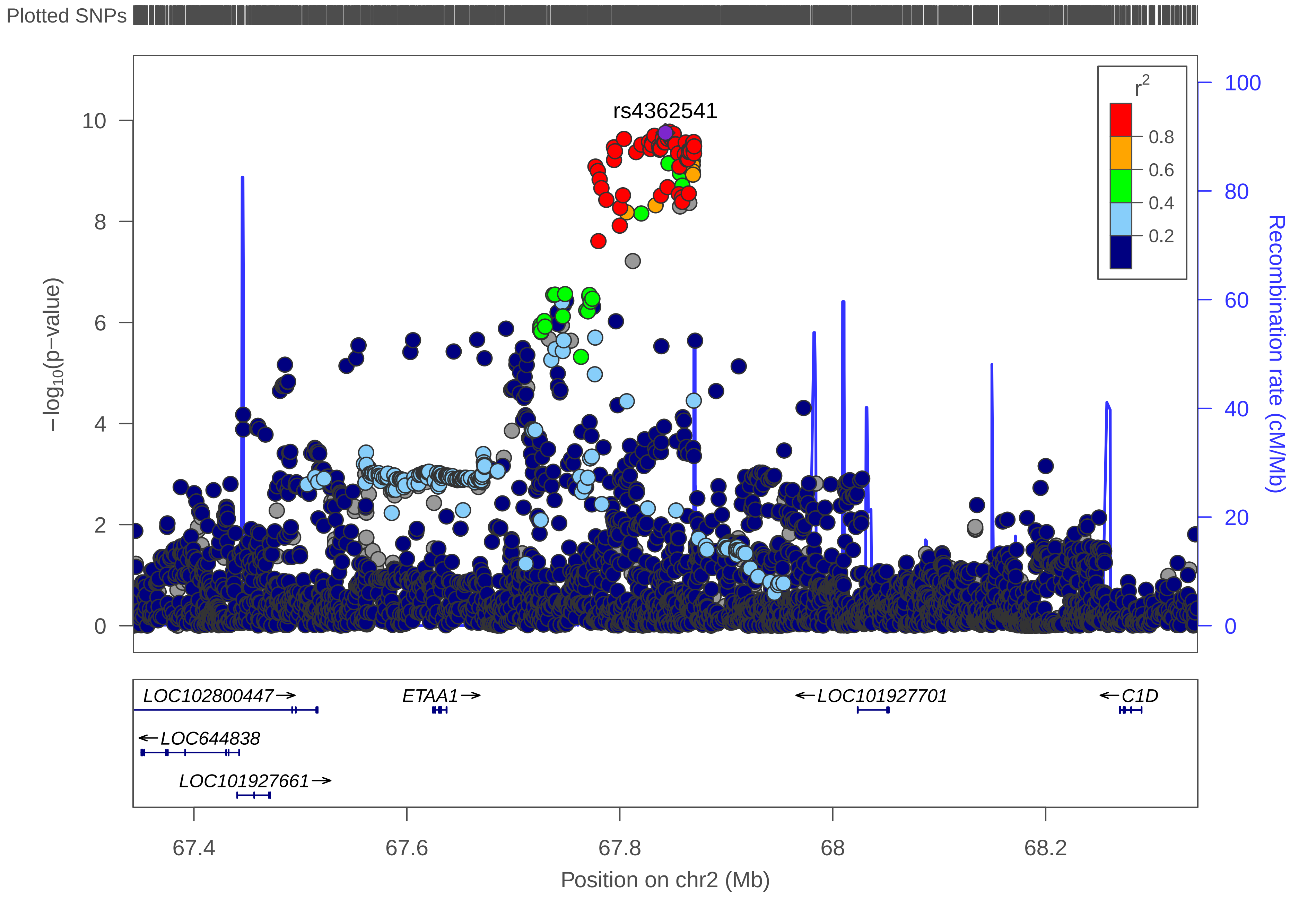
Plotted SNPs
rs4362541
r2
0.8
0.6
0.4
0.2
−log10(p−value)
Recombination rate (cM/Mb)
LOC102800447→
ETAA1→
←LOC101927701
←C1D
←LOC644838
LOC101927661→
67.4
67.6
67.8
68
68.2
Position on chr2 (Mb)

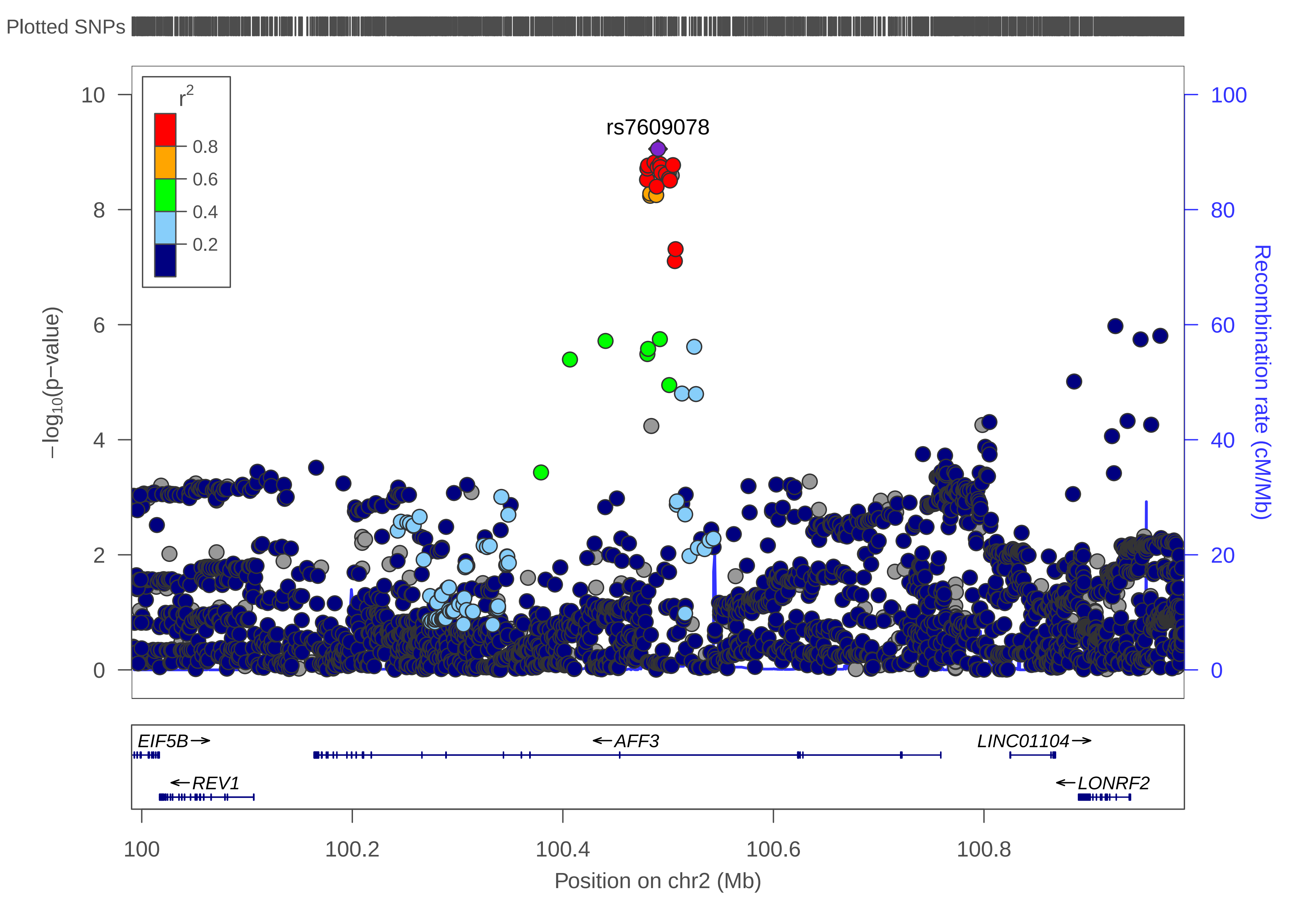

Plotted SNPs
rs7609078
r2
0.8
0.6
0.4
0.2
−log10(p−value)
Recombination rate (cM/Mb)
EIF5B→
←REV1
←AFF3
LINC01104→
←LONRF2
100
100.2
100.4
100.6
100.8
Position on chr2 (Mb)

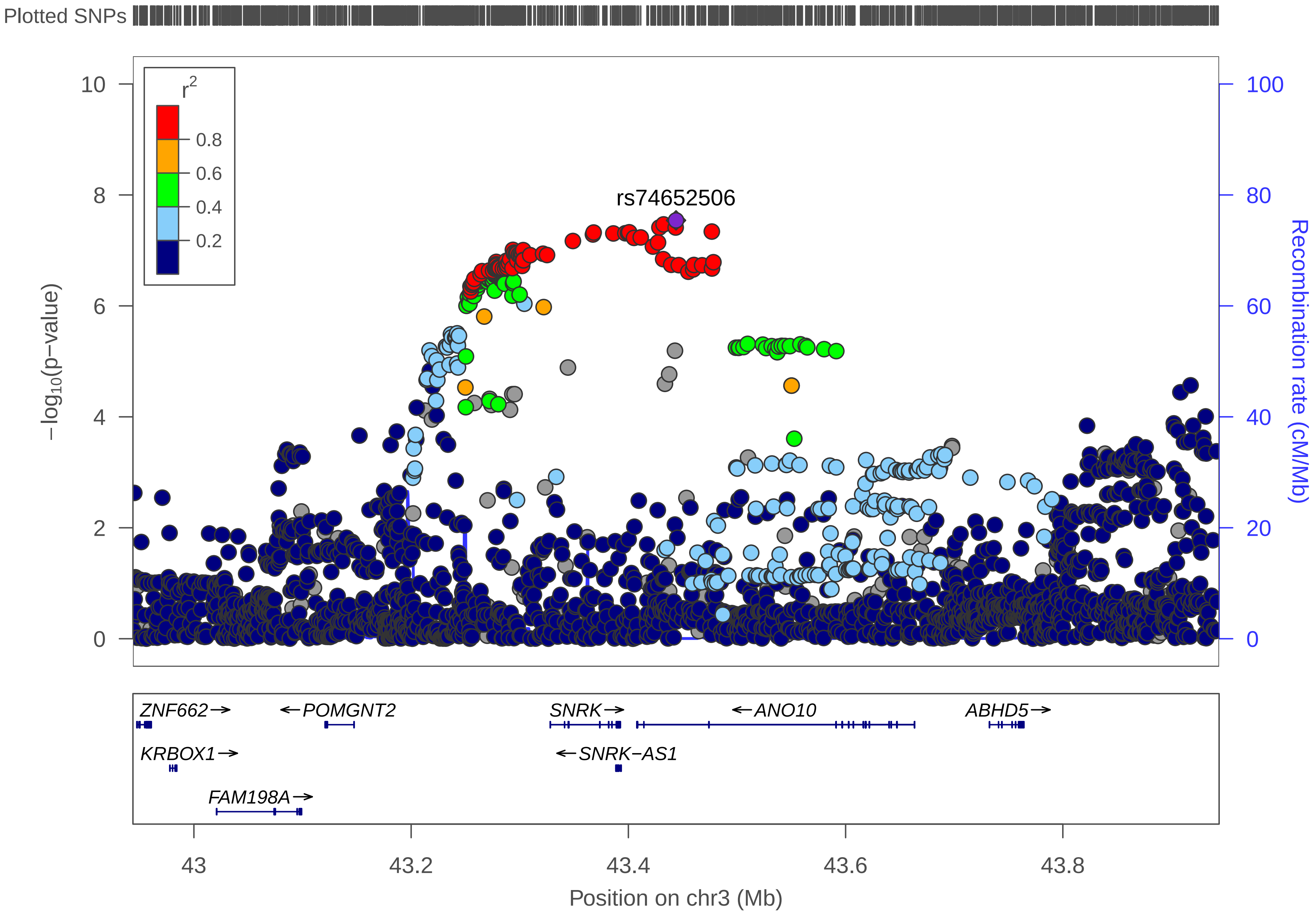

Plotted SNPs
r2
0.8
0.6
0.4
0.2
rs74652506
10
8
6
4
2
0
100
80
60
40
20
0
−log10(p−value)
Recombination rate (cM/Mb)
ZNF662→
←POMGNT2
SNRK→
←ANO10
ABHD5→
KRBOX1→
←SNRK−AS1
FAM198A→
43
43.2
43.4
43.6
43.8
Position on chr3 (Mb)

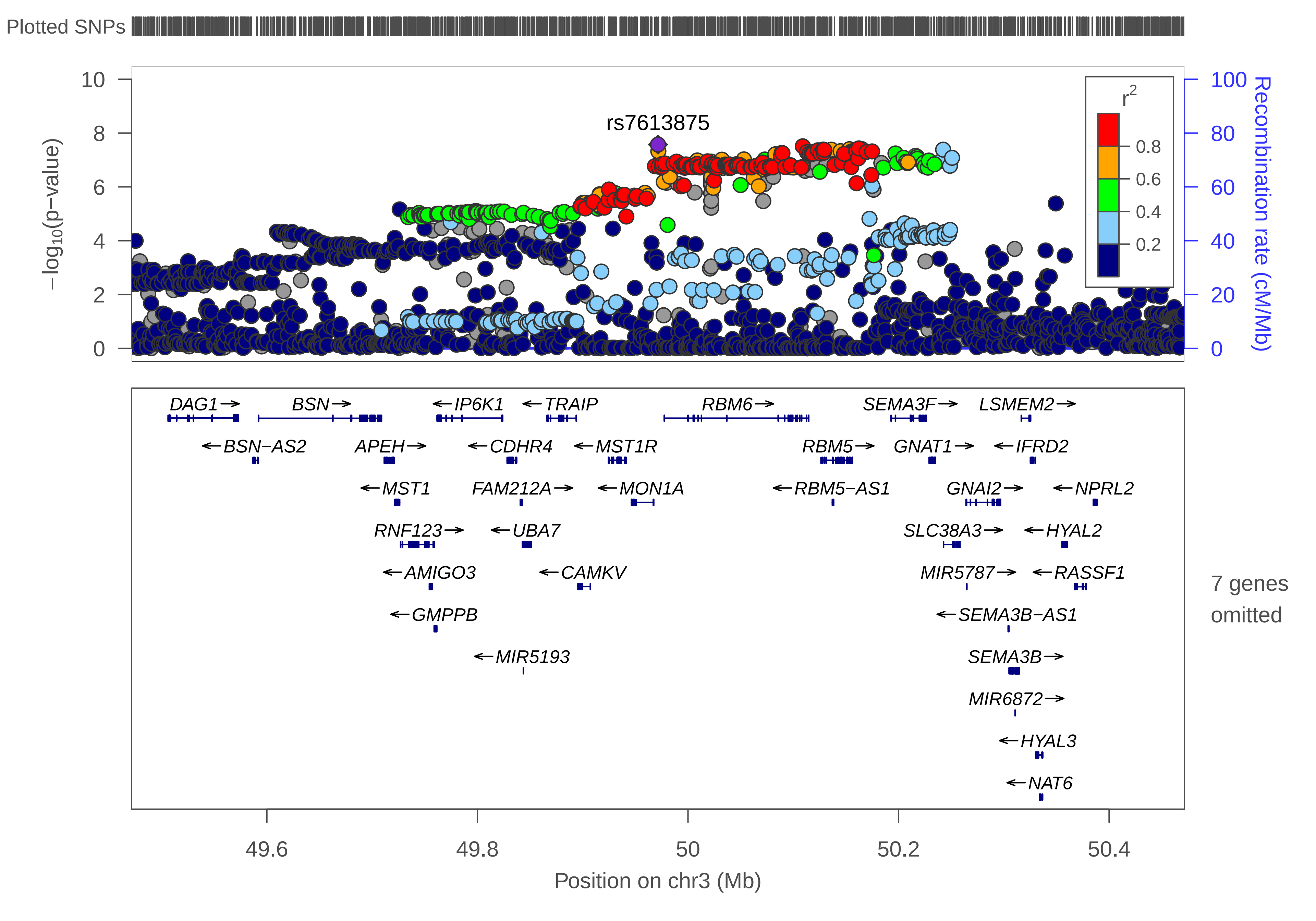

Plotted SNPs
rs7613875
−log10(p−value)
Recombination rate (cM/Mb)
r2
0.8
0.6
0.4
0.2
DAG1→
BSN→
←IP6K1
←TRAIP
RBM6→
SEMA3F→
LSMEM2→
←BSN−AS2
APEH→
←CDHR4
←MST1R
RBM5→
GNAT1→
←IFRD2
←MST1
FAM212A→
←MON1A
←RBM5−AS1
GNAI2→
←NPRL2
RNF123→
←UBA7
SLC38A3→
←HYAL2
←AMIGO3
←CAMKV
MIR5787→
←RASSF1
←GMPPB
←SEMA3B−AS1
←MIR5193
SEMA3B→
MIR6872→
←HYAL3
←NAT6
7 genes omitted
49.6
49.8
50
50.2
50.4
Position on chr3 (Mb)

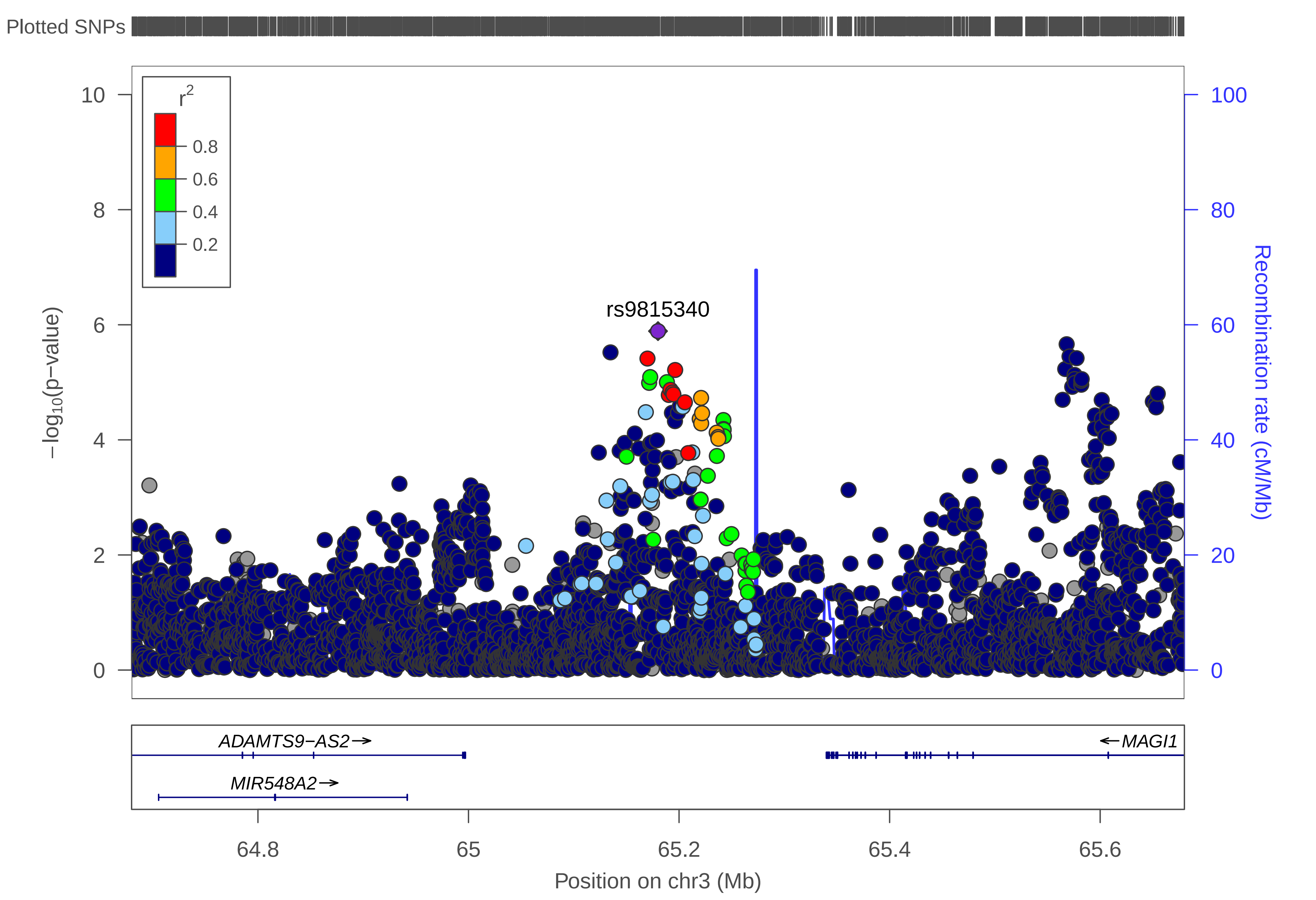
Plotted SNPs
r2
0.8
0.6
0.4
0.2
rs9815340
−log10(p−value)
Recombination rate (cM/Mb)
10
8
6
4
2
0
100
80
60
40
20
0
ADAMTS9−AS2→
MIR548A2→
←MAGI1
64.8
65
65.2
65.4
65.6
Position on chr3 (Mb)

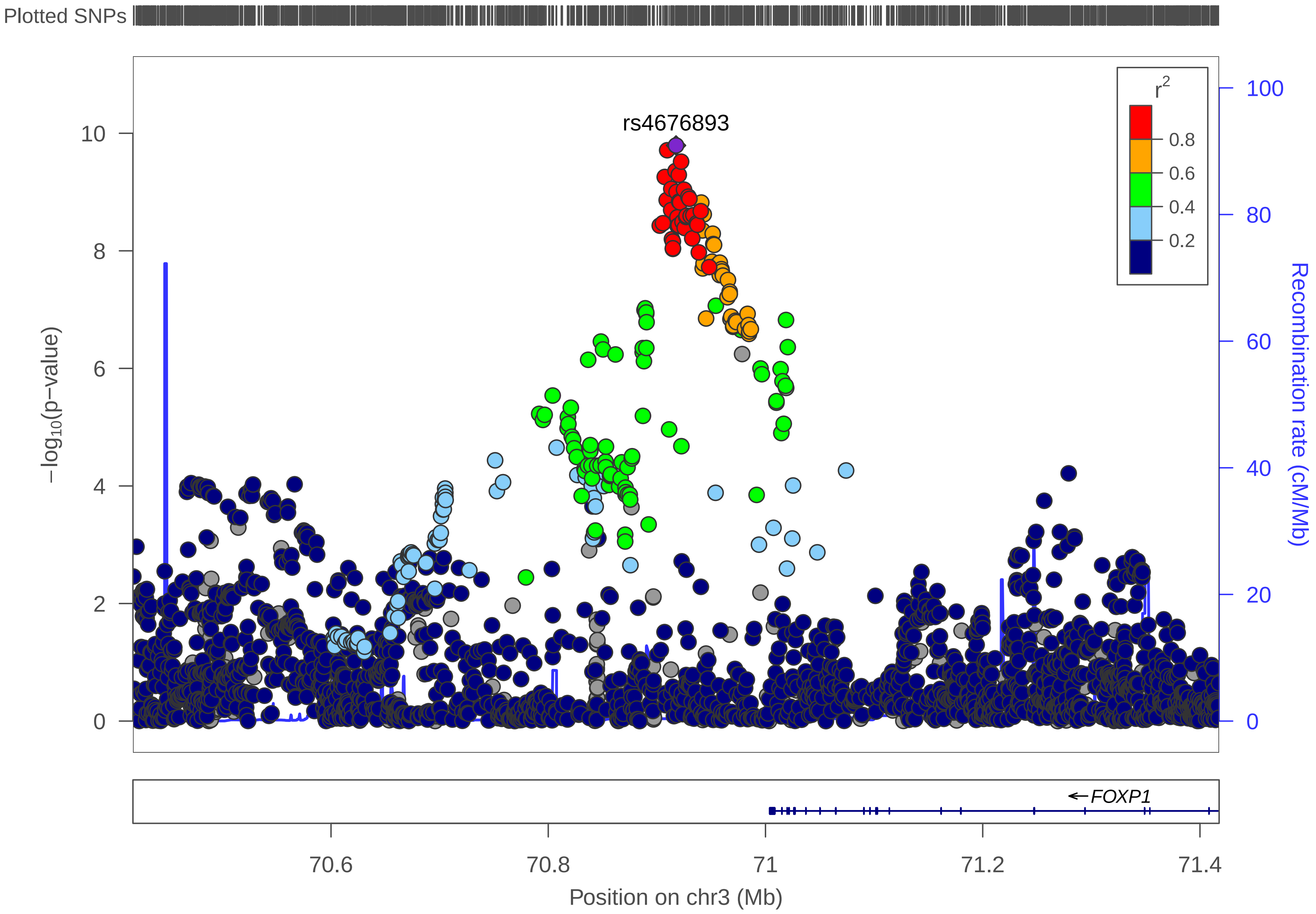
Plotted SNPs
rs4676893
r2
0.8
0.6
0.4
0.2
−log10(p−value)
Recombination rate (cM/Mb)
0
2
4
6
8
10
0
20
40
60
80
100
FOXP1
70.6
70.8
71
71.2
71.4
Position on chr3 (Mb)

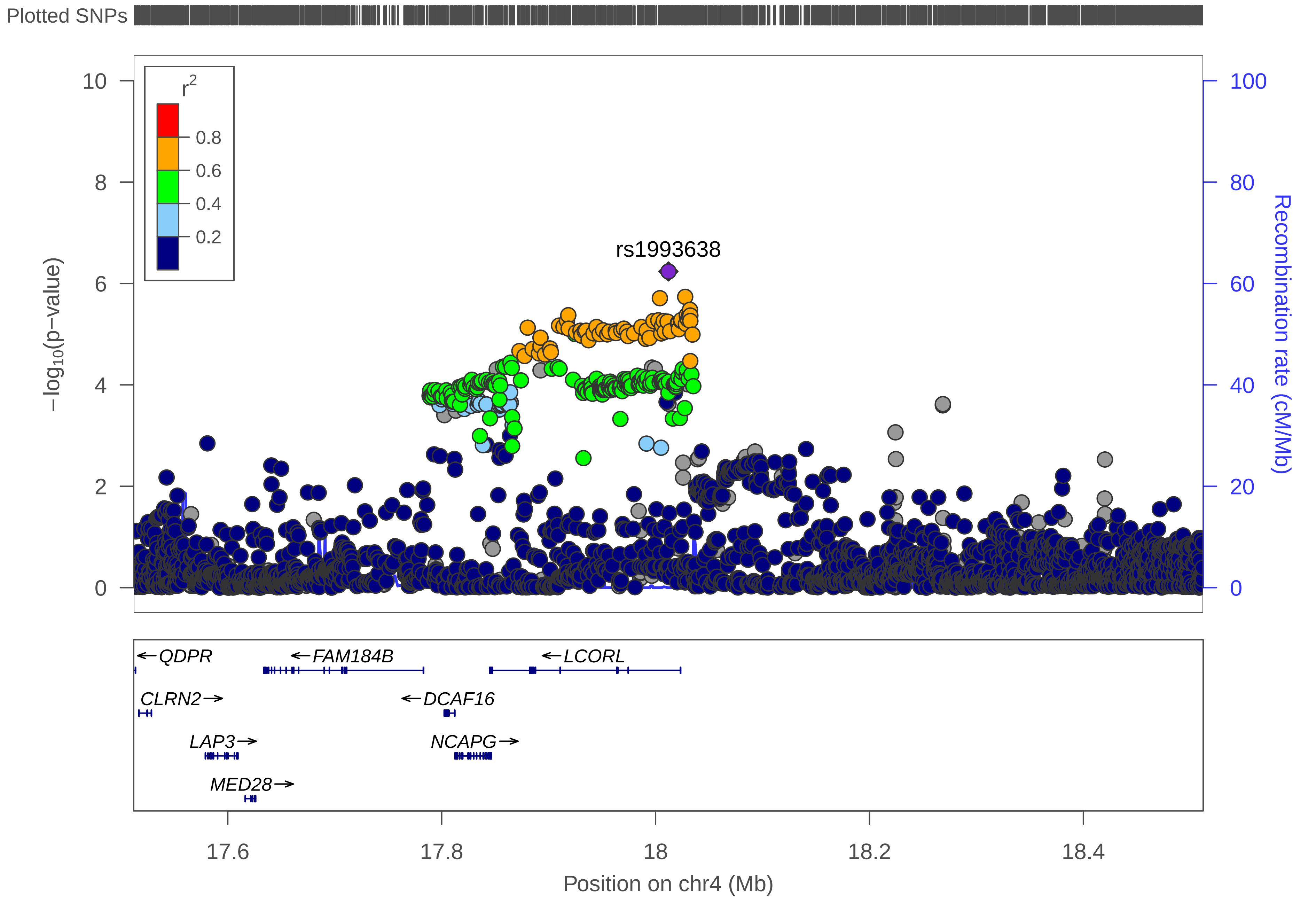

Plotted SNPs
r2
0.8
0.6
0.4
0.2
rs1993638
10
8
6
4
2
0
−log10(p−value)
100
80
60
40
20
0
Recombination rate (cM/Mb)
←QDPR
←FAM184B
←LCORL
CLRN2→
←DCAF16
LAP3→
NCAPG→
MED28→
17.6
17.8
18
18.2
18.4
Position on chr4 (Mb)

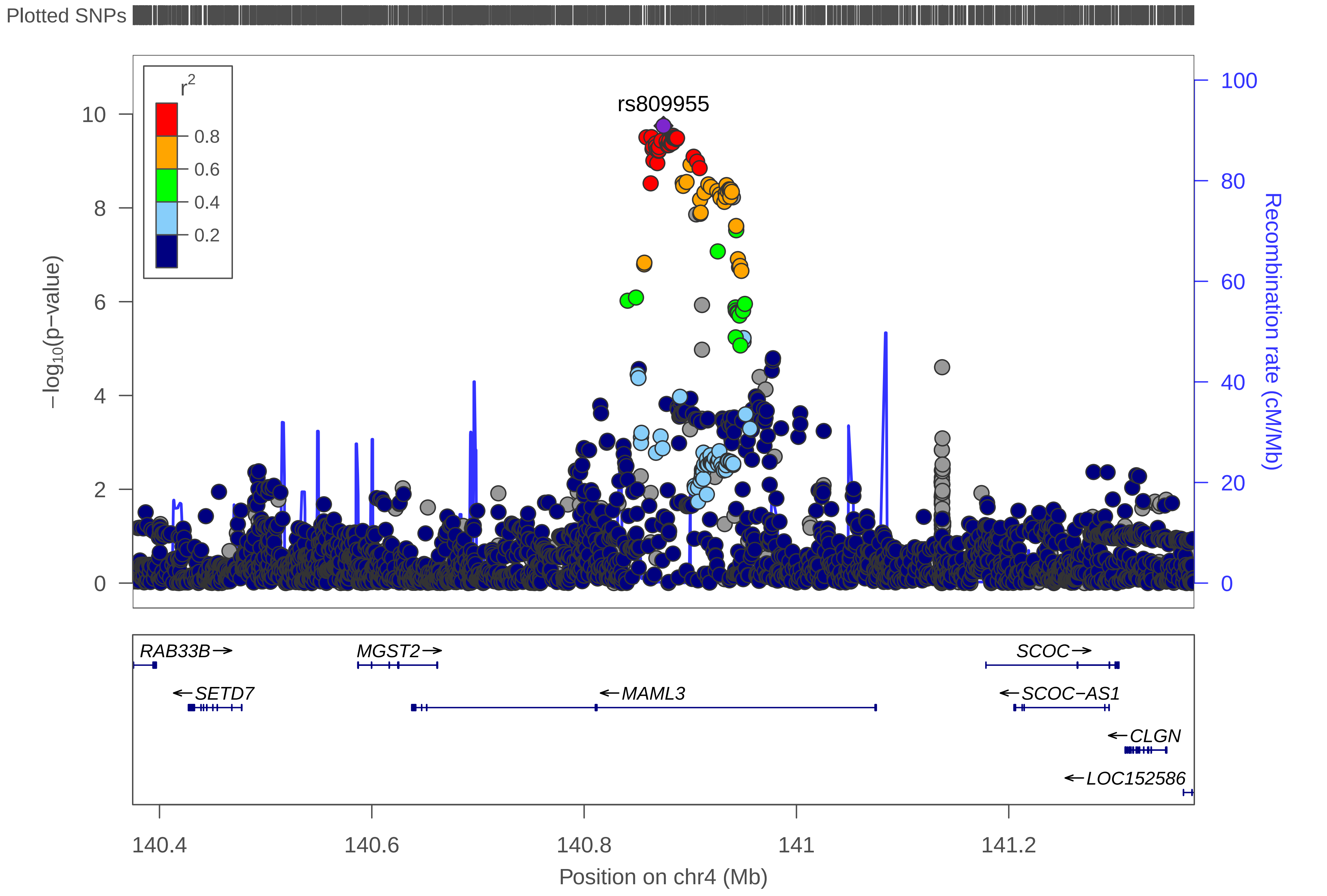
Plotted SNPs
rs809955
r²
0.8
0.6
0.4
0.2
−log10(p−value)
Recombination rate (cM/Mb)
RAB33B
SETD7
MGST2
MAML3
SCOC
SCOC−AS1
CLGN
LOC152586
140.4
140.6
140.8
141
141.2
Position on chr4 (Mb)

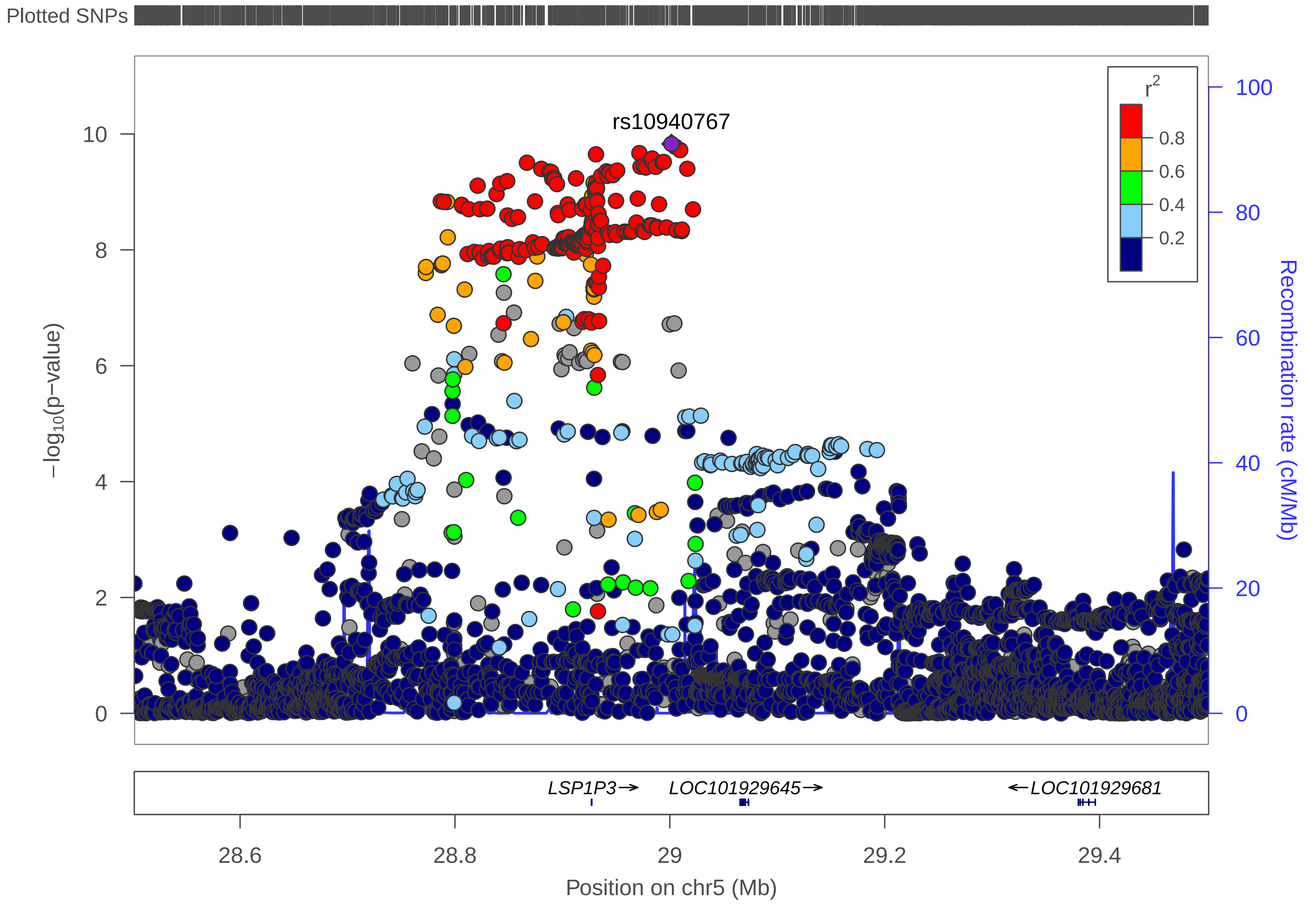

Plotted SNPs
rs10940767
r2
0.8
0.6
0.4
0.2
−log10(p−value)
0
2
4
6
8
10
Recombination rate (cM/Mb)
0
20
40
60
80
100
LSP1P3
LOC101929645
LOC101929681
28.6
28.8
29
29.2
29.4
Position on chr5 (Mb)

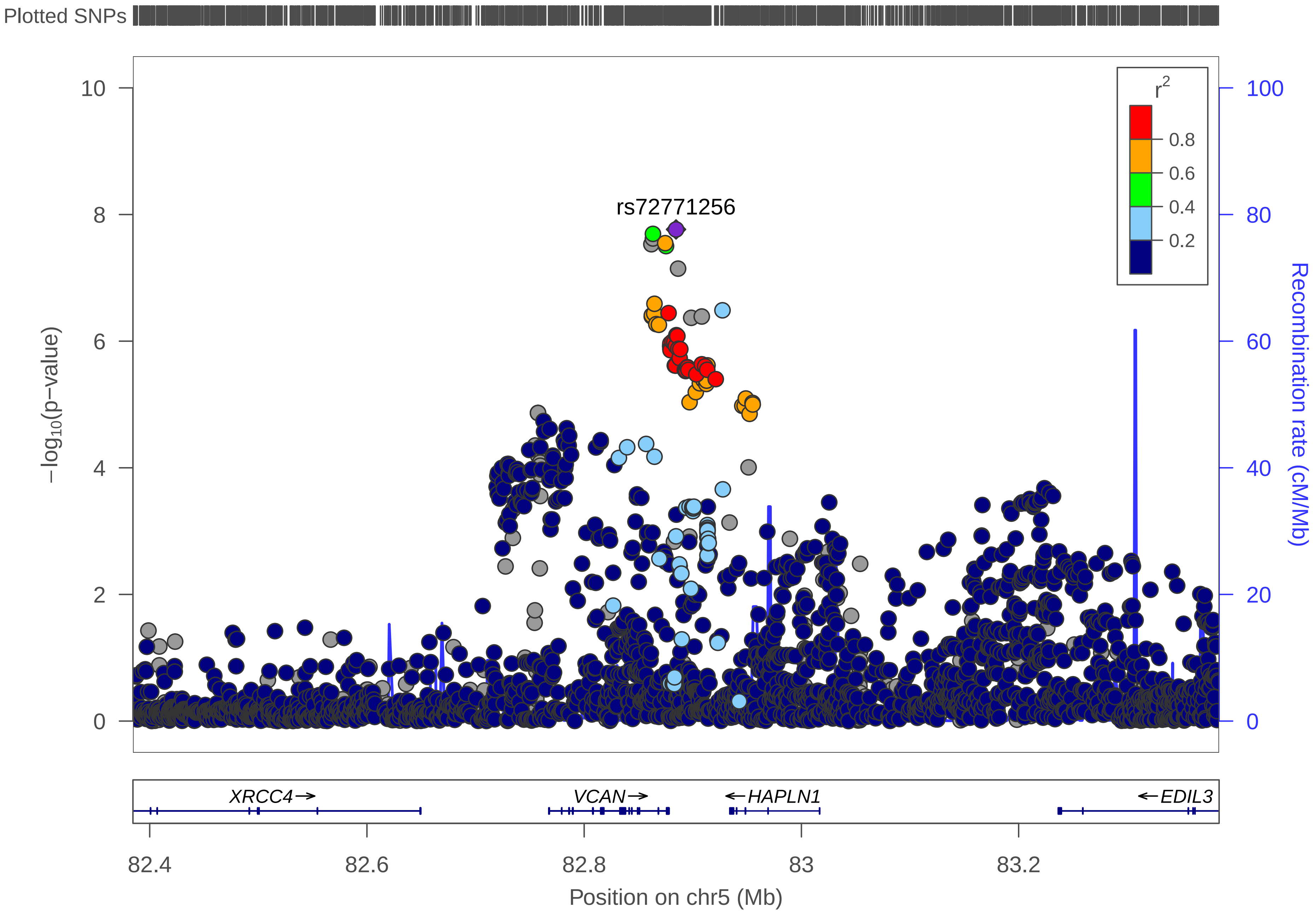
Plotted SNPs
rs72771256
r2
0.8
0.6
0.4
0.2
10
8
6
4
2
0
−log10(p−value)
100
80
60
40
20
0
Recombination rate (cM/Mb)
XRCC4→
VCAN→
←HAPLN1
←EDIL3
82.4
82.6
82.8
83
83.2
Position on chr5 (Mb)

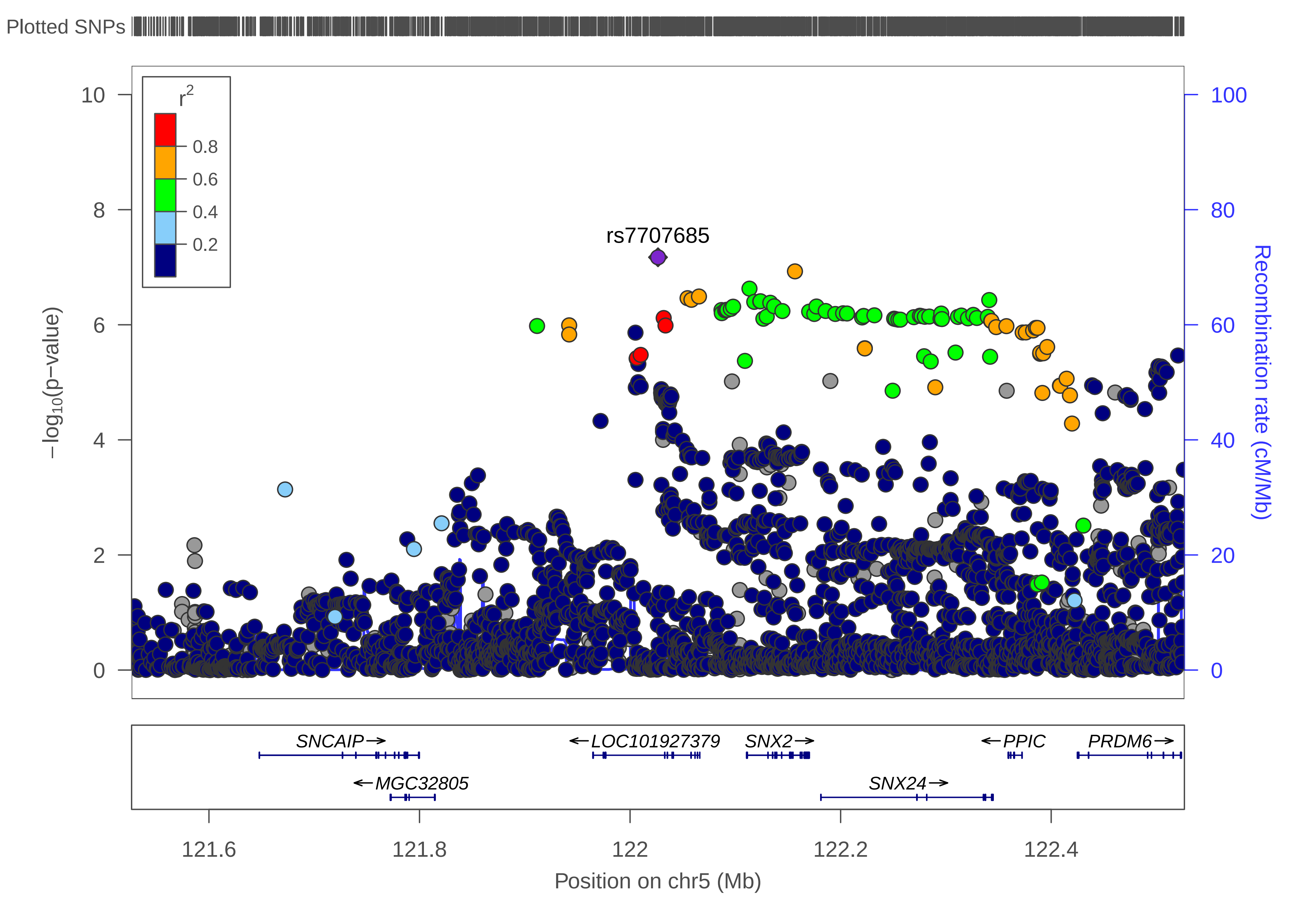
Plotted SNPs
r2
0.8
0.6
0.4
0.2
rs7707685
−log10(p−value)
Recombination rate (cM/Mb)
0
2
4
6
8
10
0
20
40
60
80
100
SNCAIP→
←MGC32805
←LOC101927379
SNX2→
SNX24→
←PPIC
PRDM6→
121.6
121.8
122
122.2
122.4
Position on chr5 (Mb)

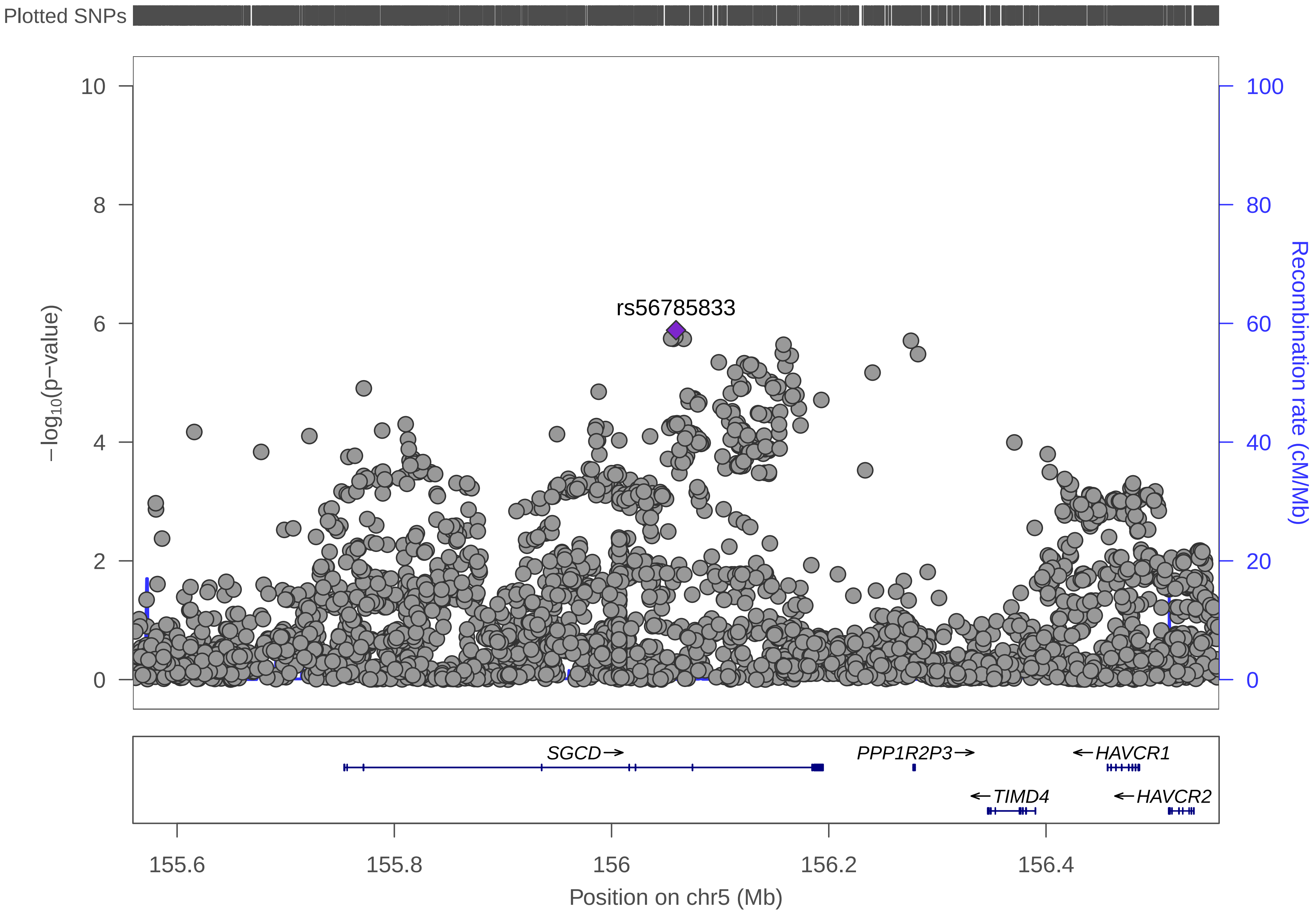

Plotted SNPs
−log10(p−value)
Recombination rate (cM/Mb)
rs56785833
SGCD→
PPP1R2P3→
←HAVCR1
←TIMD4
←HAVCR2
155.6
155.8
156
156.2
156.4
Position on chr5 (Mb)

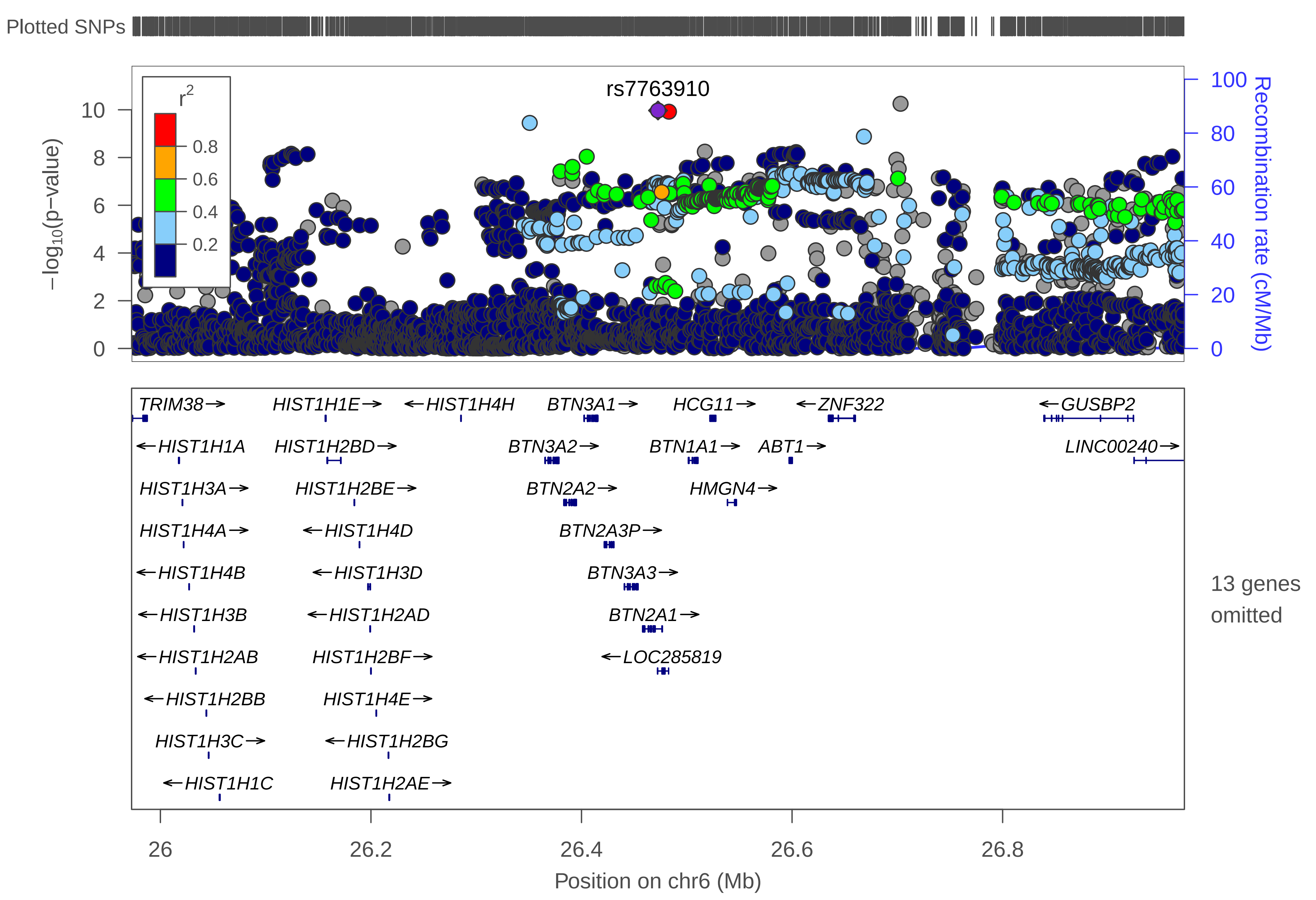

Plotted SNPs
rs7763910
r2
0.8
0.6
0.4
0.2
−log10(p−value)
Recombination rate (cM/Mb)
0
2
4
6
8
10
20
40
60
80
100
TRIM38→
HIST1H1E→
←HIST1H4H
BTN3A1→
HCG11→
←ZNF322
←GUSBP2
←HIST1H1A
HIST1H2BD→
BTN3A2→
BTN1A1→
ABT1→
LINC00240→
HIST1H3A→
HIST1H2BE→
BTN2A2→
HMGN4→
HIST1H4A→
←HIST1H4D
BTN2A3P→
←HIST1H4B
←HIST1H3D
BTN3A3→
←HIST1H3B
←HIST1H2AD
BTN2A1→
←HIST1H2AB
HIST1H2BF→
←LOC285819
←HIST1H2BB
HIST1H4E→
HIST1H3C→
←HIST1H2BG
←HIST1H1C
HIST1H2AE→
13 genes omitted
26
26.2
26.4
26.6
26.8
Position on chr6 (Mb)

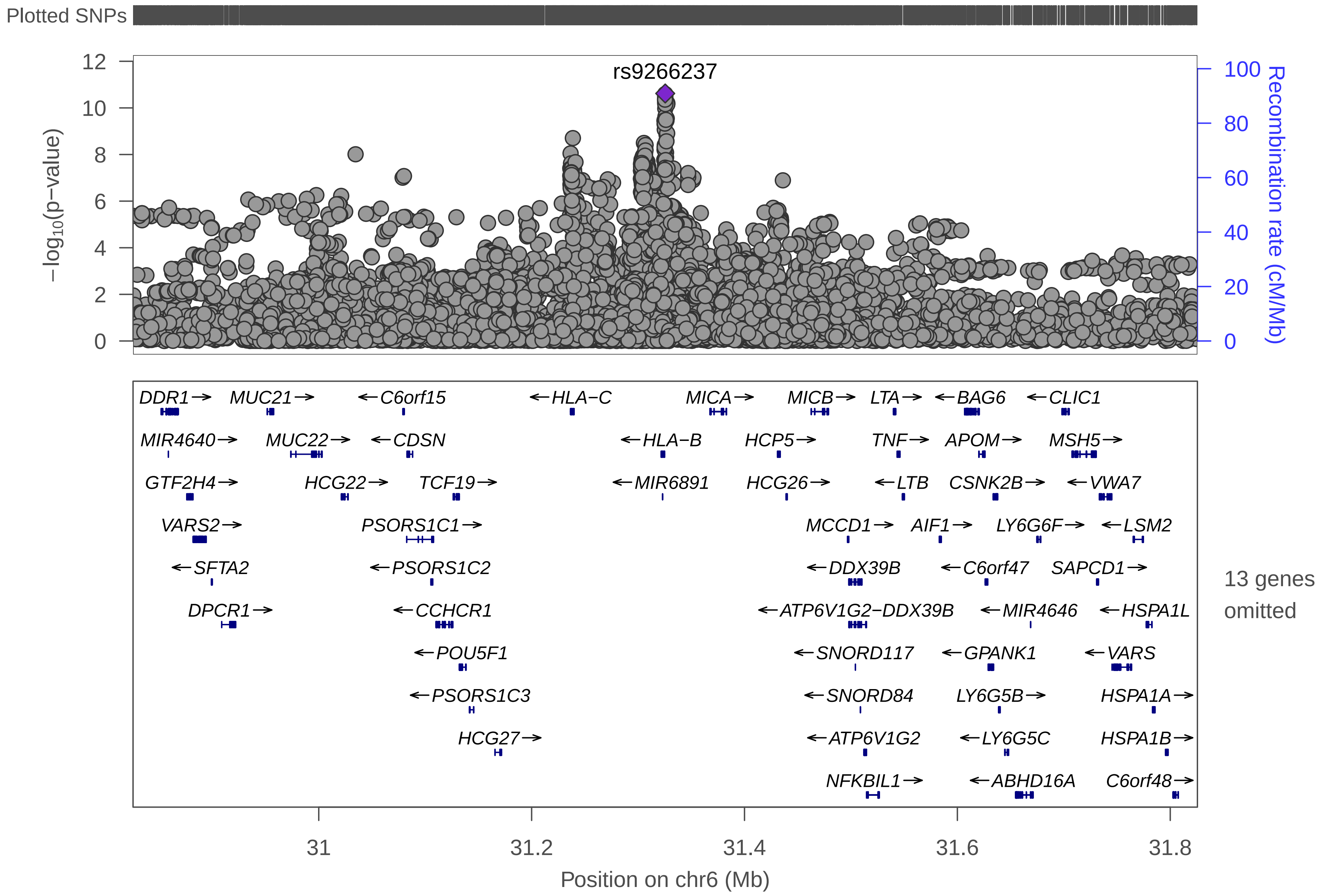
Plotted SNPs
rs9266237
−log10(p−value)
Recombination rate (cM/Mb)
0
2
4
6
8
10
12
0
20
40
60
80
100
DDR1→
MUC21→
←C6orf15
←HLA−C
MICA→
MICB→
LTA→
←BAG6
←CLIC1
MIR4640→
MUC22→
←CDSN
←HLA−B
HCP5→
TNF→
APOM→
MSH5→
GTF2H4→
HCG22→
TCF19→
←MIR6891
HCG26→
←LTB
CSNK2B→
←VWA7
VARS2→
PSORS1C1→
MCCD1→
AIF1→
LY6G6F→
←LSM2
←SFTA2
←PSORS1C2
←DDX39B
←C6orf47
SAPCD1→
DPCR1→
←CCHCR1
←ATP6V1G2−DDX39B
←MIR4646
←HSPA1L
←POU5F1
←SNORD117
←GPANK1
←VARS
←PSORS1C3
←SNORD84
LY6G5B→
HSPA1A→
HCG27→
←ATP6V1G2
←LY6G5C
HSPA1B→
NFKBIL1→
←ABHD16A
C6orf48→
13 genes omitted
31
31.2
31.4
31.6
31.8
Position on chr6 (Mb)

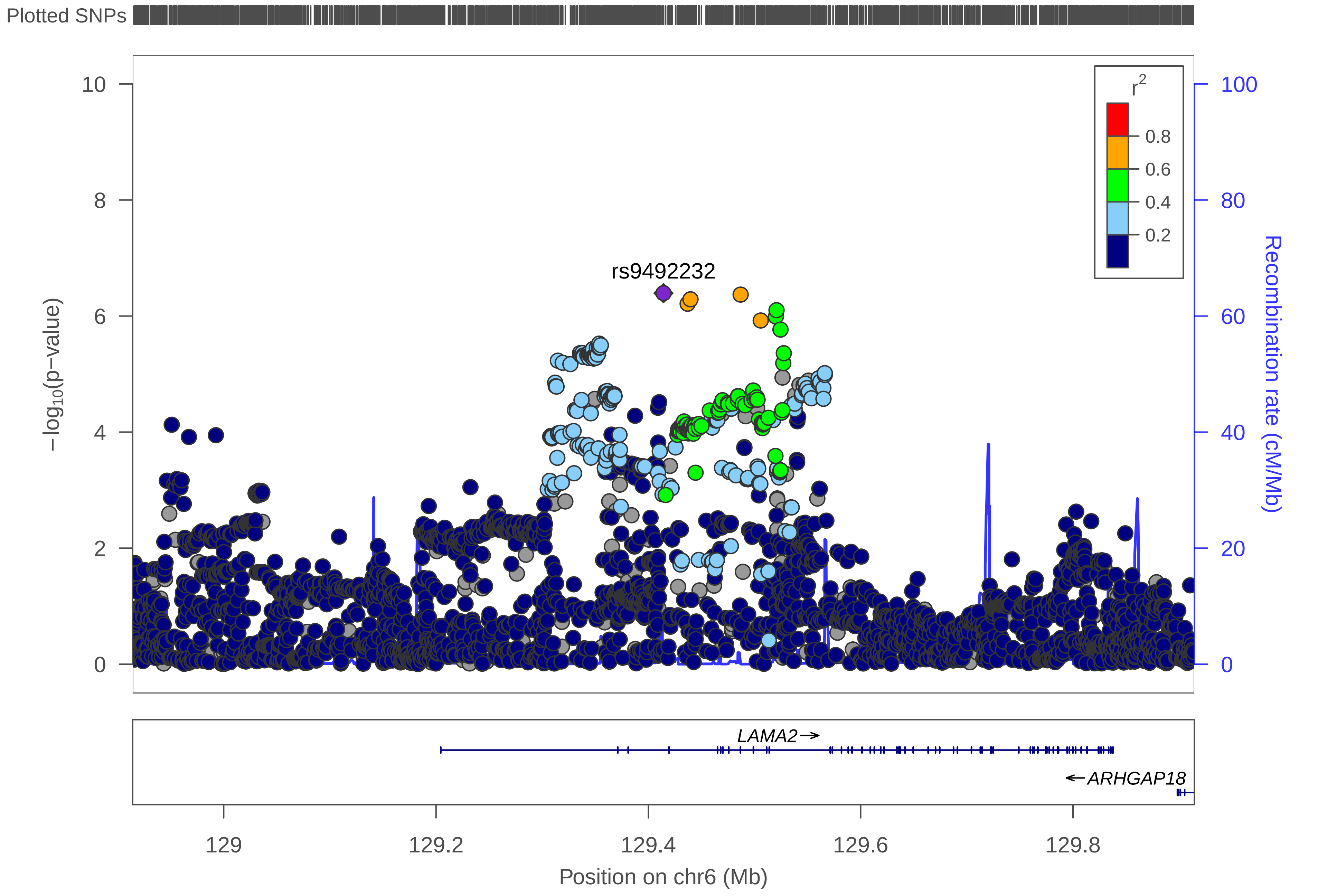

Plotted SNPs
rs9492232
r^2
0.8
0.6
0.4
0.2
10
8
6
4
2
0
100
80
60
40
20
0
−log10(p−value)
Recombination rate (cM/Mb)
LAMA2→
←ARHGAP18
129
129.2
129.4
129.6
129.8
Position on chr6 (Mb)

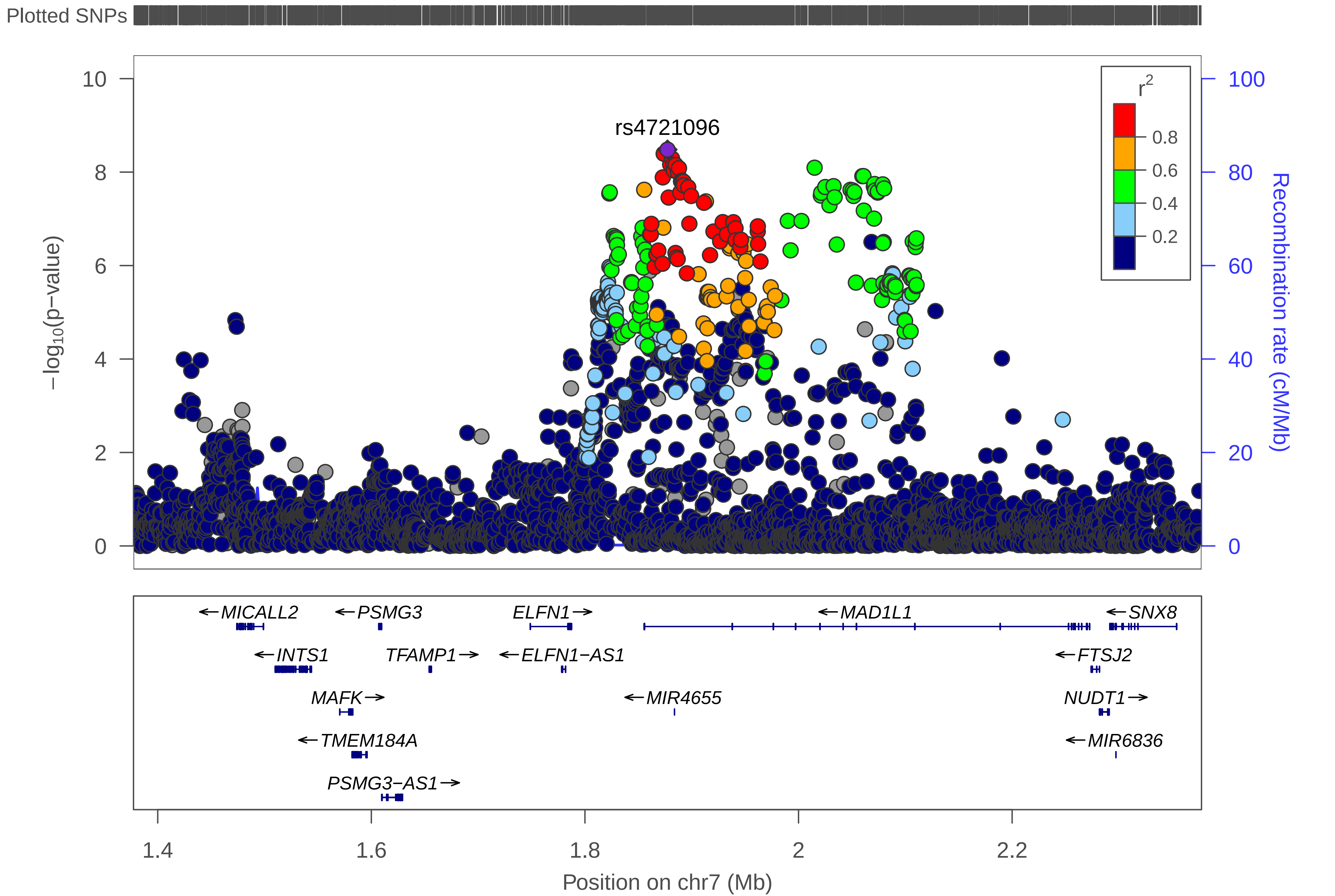
Plotted SNPs
rs4721096
r2
0.8
0.6
0.4
0.2
−log10(p−value)
Recombination rate (cM/Mb)
10
8
6
4
2
0
100
80
60
40
20
0
←MICALL2
←PSMG3
ELFN1→
←MAD1L1
←SNX8
←INTS1
TFAMP1→
←ELFN1−AS1
←FTSJ2
MAFK→
←MIR4655
NUDT1→
←TMEM184A
←MIR6836
PSMG3−AS1→
1.4
1.6
1.8
2
2.2
Position on chr7 (Mb)

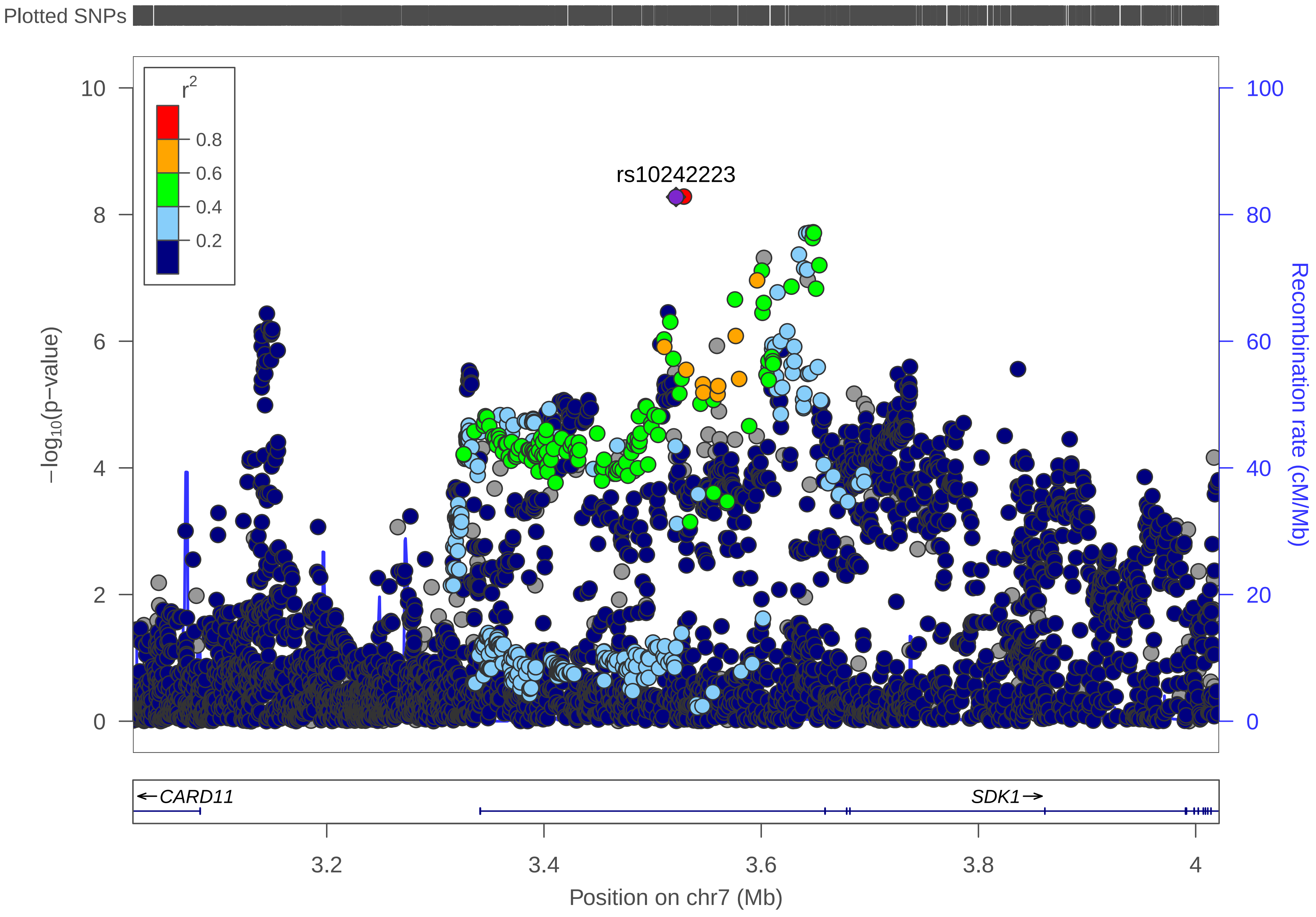
Plotted SNPs
r2
0.8
0.6
0.4
0.2
rs10242223
10
8
6
4
2
0
−log10(p−value)
100
80
60
40
20
0
Recombination rate (cM/Mb)
←CARD11
SDK1→
3.2
3.4
3.6
3.8
4
Position on chr7 (Mb)

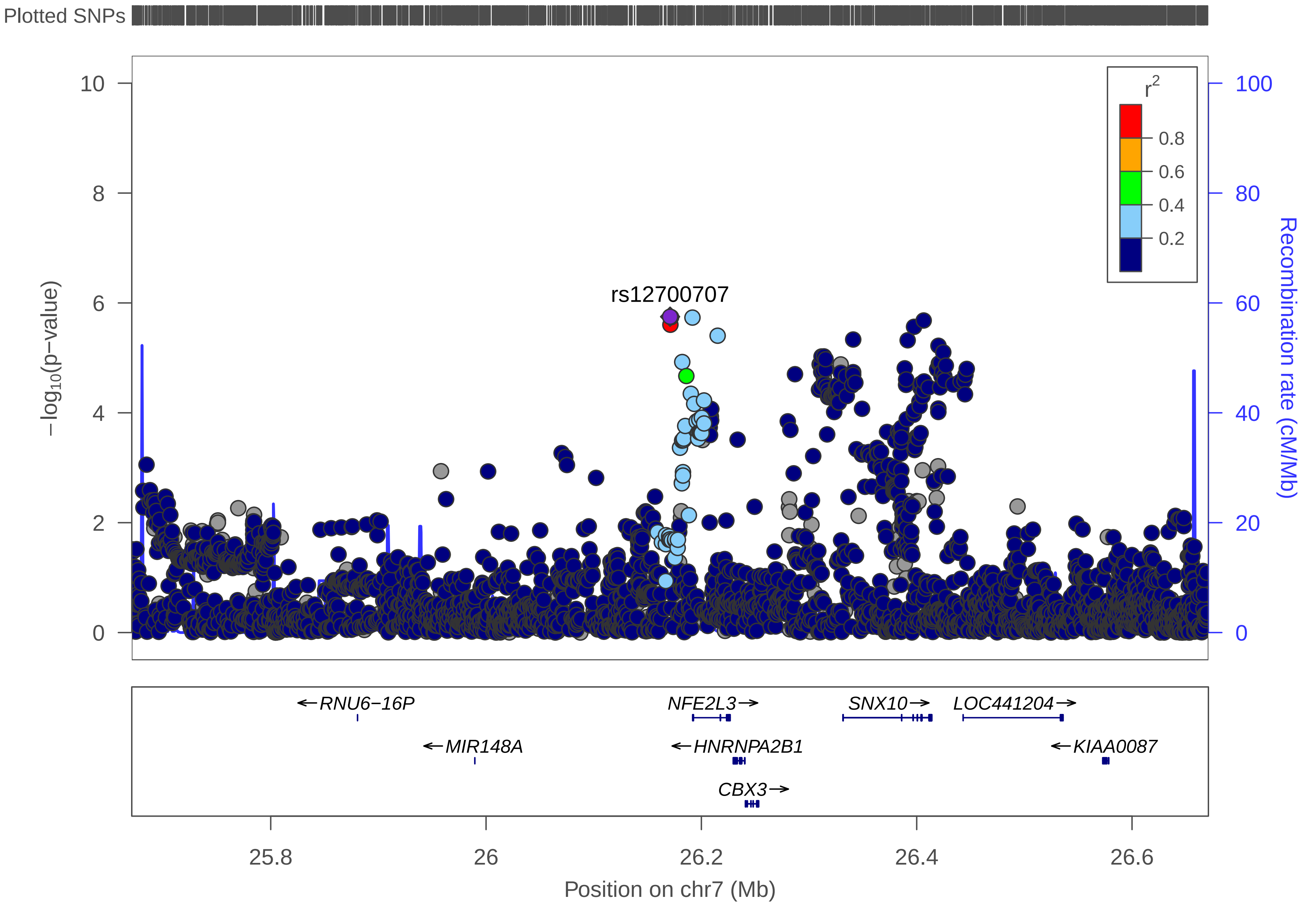

Plotted SNPs
rs12700707
r2
0.8
0.6
0.4
0.2
−log10(p−value)
Recombination rate (cM/Mb)
10
8
6
4
2
0
100
80
60
40
20
0
←RNU6−16P
NFE2L3→
SNX10→
LOC441204→
←MIR148A
←HNRNPA2B1
←KIAA0087
CBX3→
25.8
26
26.2
26.4
26.6
Position on chr7 (Mb)

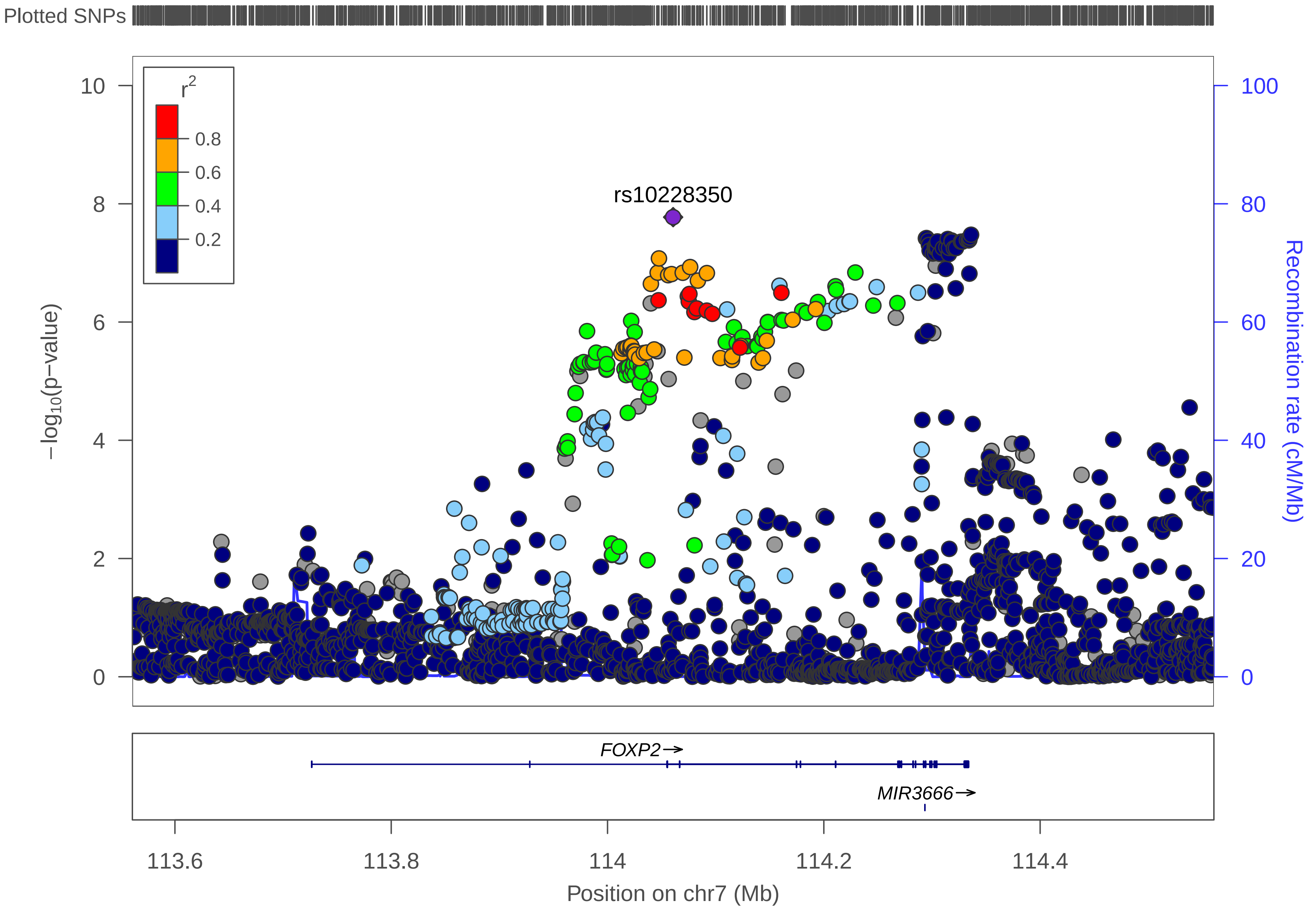

Plotted SNPs
r2
0.8
0.6
0.4
0.2
rs10228350
−log10(p−value)
Recombination rate (cM/Mb)
10
8
6
4
2
0
100
80
60
40
20
0
FOXP2→
MIR3666→
113.6
113.8
114
114.2
114.4
Position on chr7 (Mb)

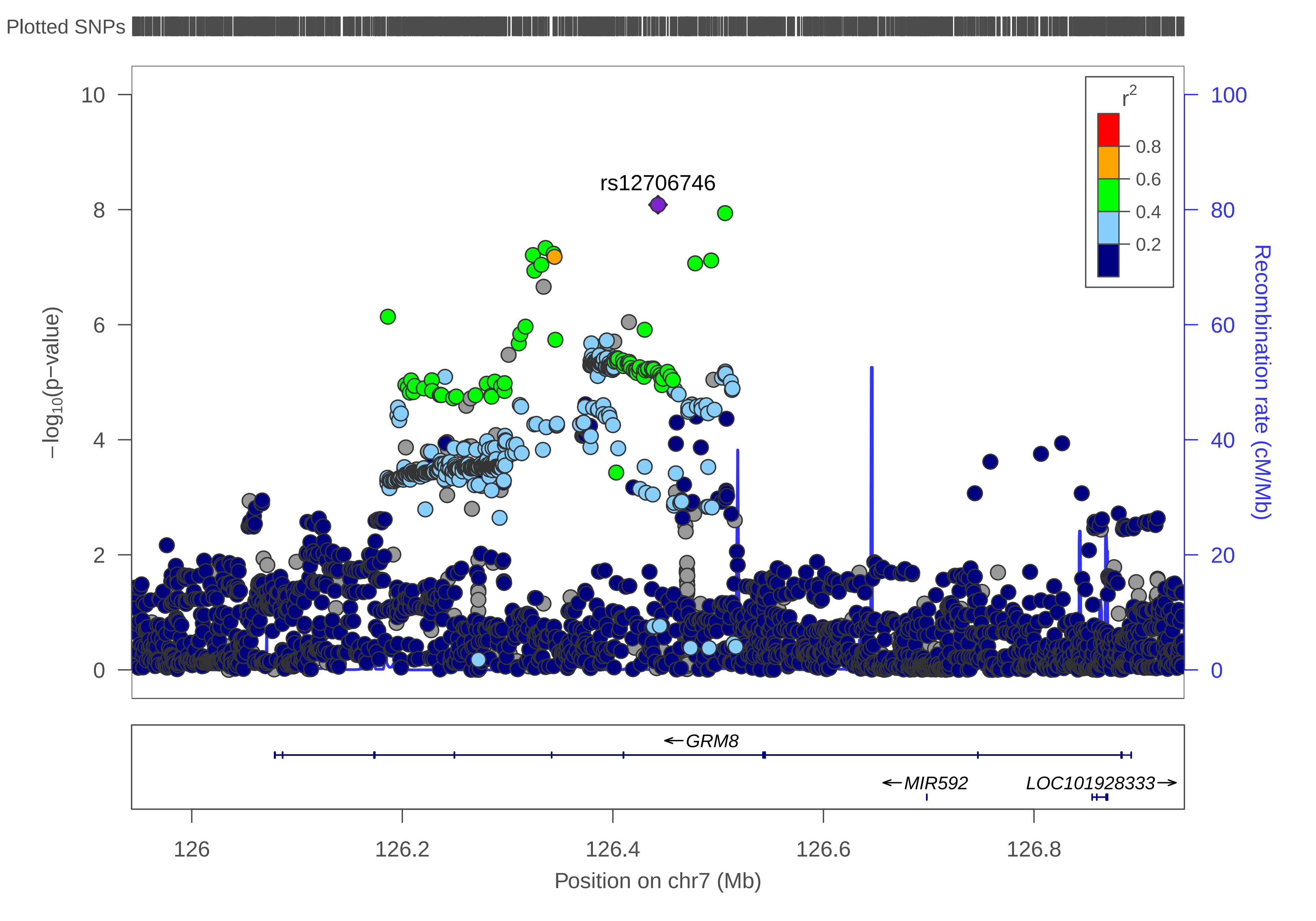
Plotted SNPs
rs12706746
r^2
0.8
0.6
0.4
0.2
−log10(p−value)
Recombination rate (cM/Mb)
0
2
4
6
8
10
0
20
40
60
80
100
← GRM8
← MIR592
LOC101928333 →
126
126.2
126.4
126.6
126.8
Position on chr7 (Mb)

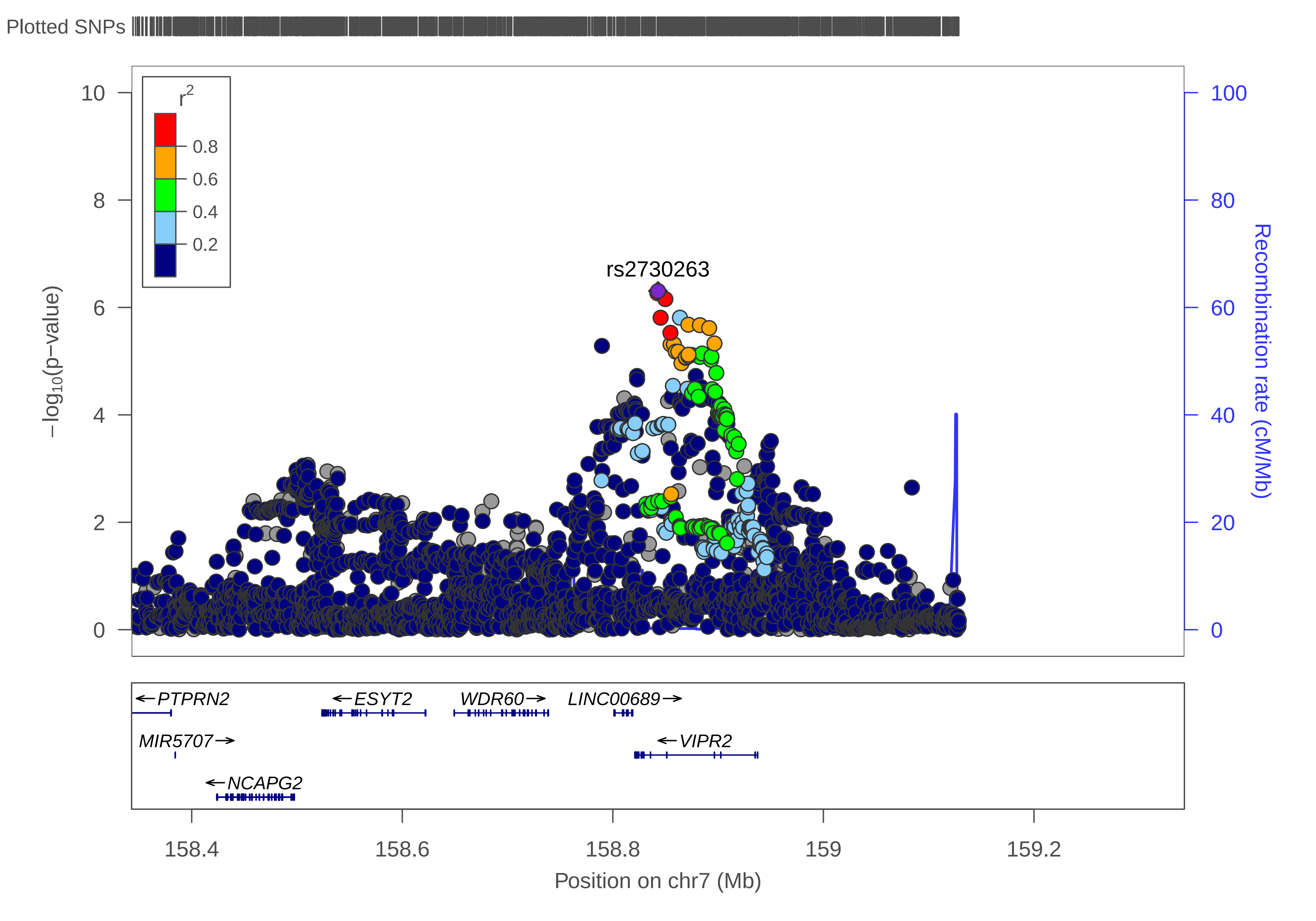

Plotted SNPs
r2
0.8
0.6
0.4
0.2
rs2730263
−log10(p−value)
Recombination rate (cM/Mb)
10
8
6
4
2
0
100
80
60
40
20
0
PTPRN2
ESYT2
WDR60
LINC00689
MIR5707
VIPR2
NCAPG2
158.4
158.6
158.8
159
159.2
Position on chr7 (Mb)

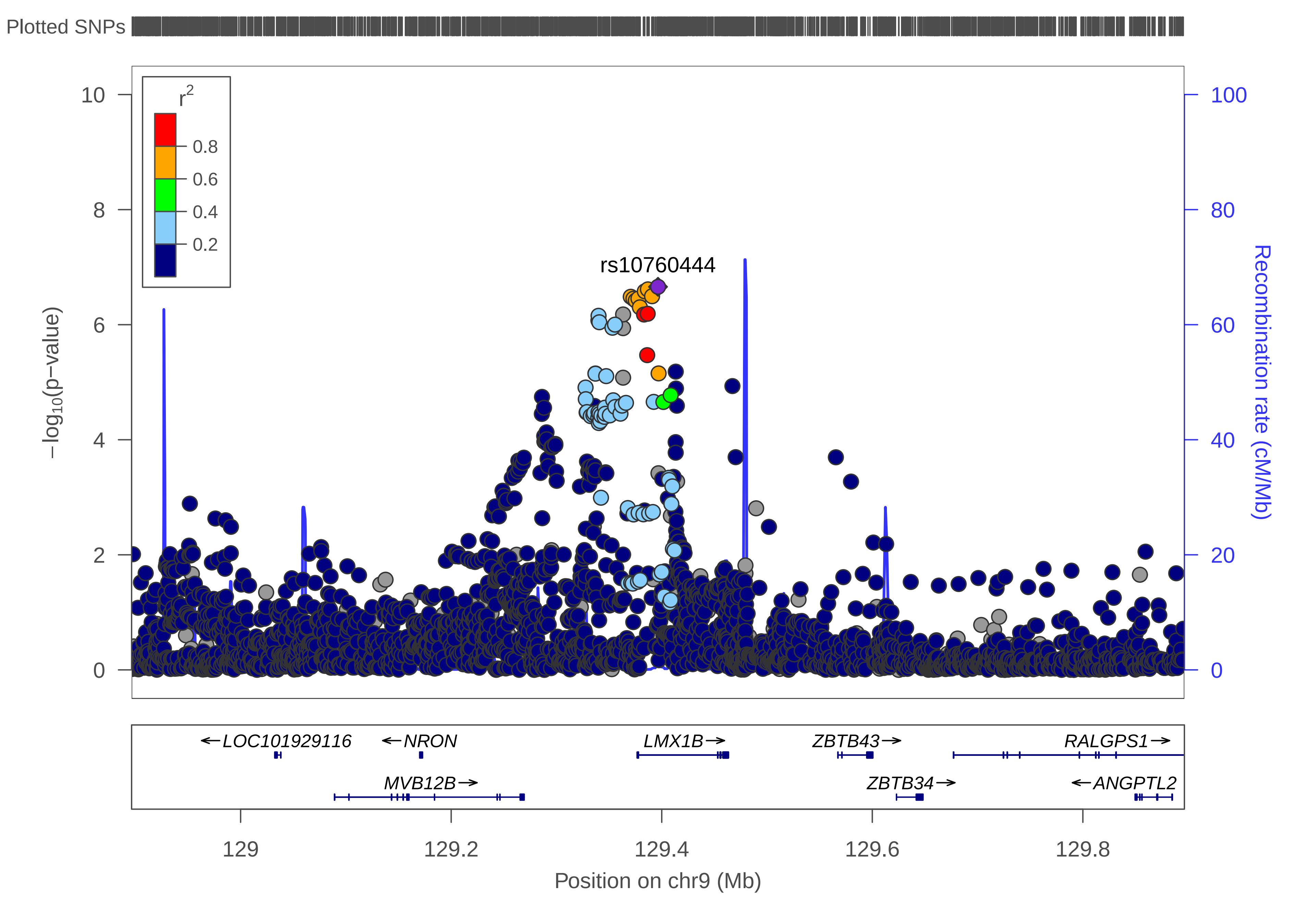
Plotted SNPs
r2
0.8
0.6
0.4
0.2
rs10760444
−log10(p−value)
Recombination rate (cM/Mb)
0
2
4
6
8
10
0
20
40
60
80
100
←LOC101929116
←NRON
LMX1B→
ZBTB43→
RALGPS1→
MVB12B→
ZBTB34→
←ANGPTL2
129
129.2
129.4
129.6
129.8
Position on chr9 (Mb)

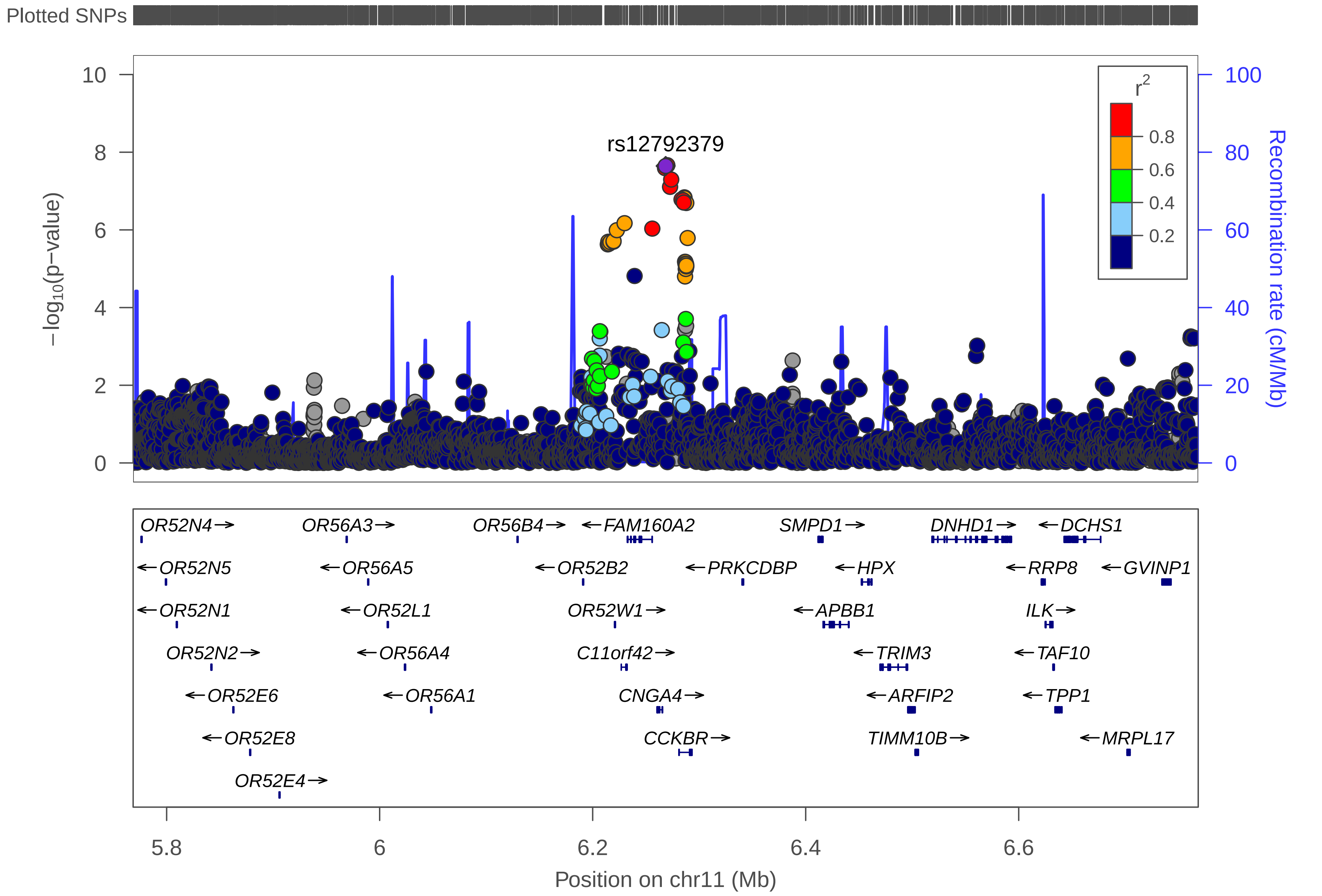
Plotted SNPs
rs12792379
−log10(p−value)
Recombination rate (cM/Mb)
r2
0.8
0.6
0.4
0.2
10
8
6
4
2
0
100
80
60
40
20
0
OR52N4→
OR56A3→
OR56B4→
←FAM160A2
SMPD1→
DNHD1→
←DCHS1
←OR52N5
←OR56A5
←OR52B2
←PRKCDBP
←HPX
←RRP8
←GVINP1
←OR52N1
←OR52L1
OR52W1→
←APBB1
ILK→
OR52N2→
←OR56A4
C11orf42→
←TRIM3
←TAF10
←OR52E6
←OR56A1
CNGA4→
←ARFIP2
←TPP1
←OR52E8
CCKBR→
TIMM10B→
←MRPL17
OR52E4→
5.8
6
6.2
6.4
6.6
Position on chr11 (Mb)

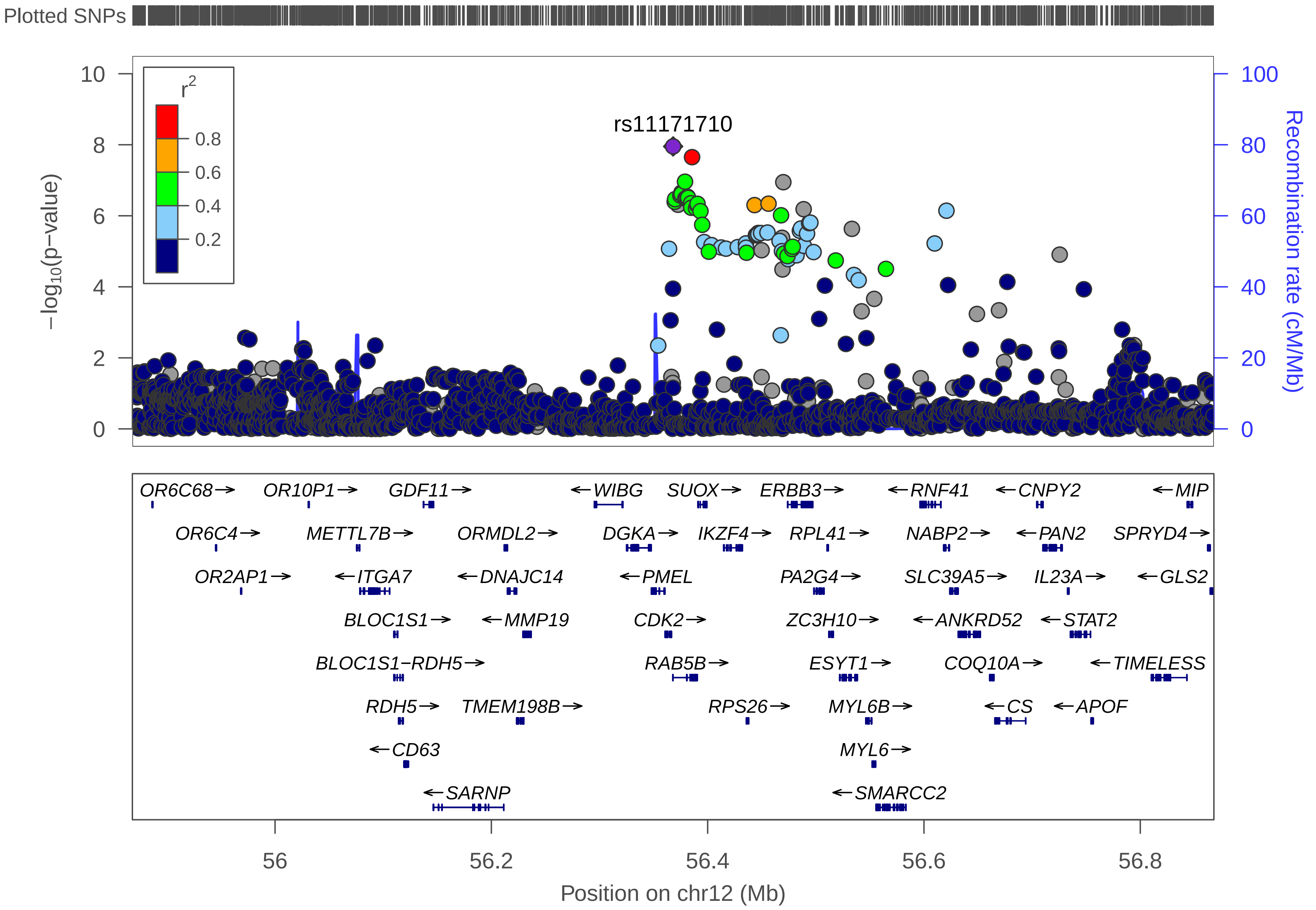

Plotted SNPs
rs11171710
r2
0.8
0.6
0.4
0.2
−log10(p−value)
Recombination rate (cM/Mb)
0
2
4
6
8
10
20
40
60
80
100
OR6C68→
OR10P1→
GDF11→
←WIBG
SUOX→
ERBB3→
←RNF41
←CNPY2
←MIP
OR6C4→
METTL7B→
ORMDL2→
DGKA→
IKZF4→
RPL41→
NABP2→
←PAN2
SPRYD4→
OR2AP1→
←ITGA7
←DNAJC14
←PMEL
PA2G4→
SLC39A5→
IL23A→
←GLS2
BLOC1S1→
←MMP19
CDK2→
ZC3H10→
←ANKRD52
←STAT2
BLOC1S1−RDH5→
RAB5B→
ESYT1→
COQ10A→
←TIMELESS
RDH5→
TMEM198B→
RPS26→
MYL6B→
←CS
←APOF
←CD63
MYL6→
←SARNP
←SMARCC2
56
56.2
56.4
56.6
56.8
Position on chr12 (Mb)

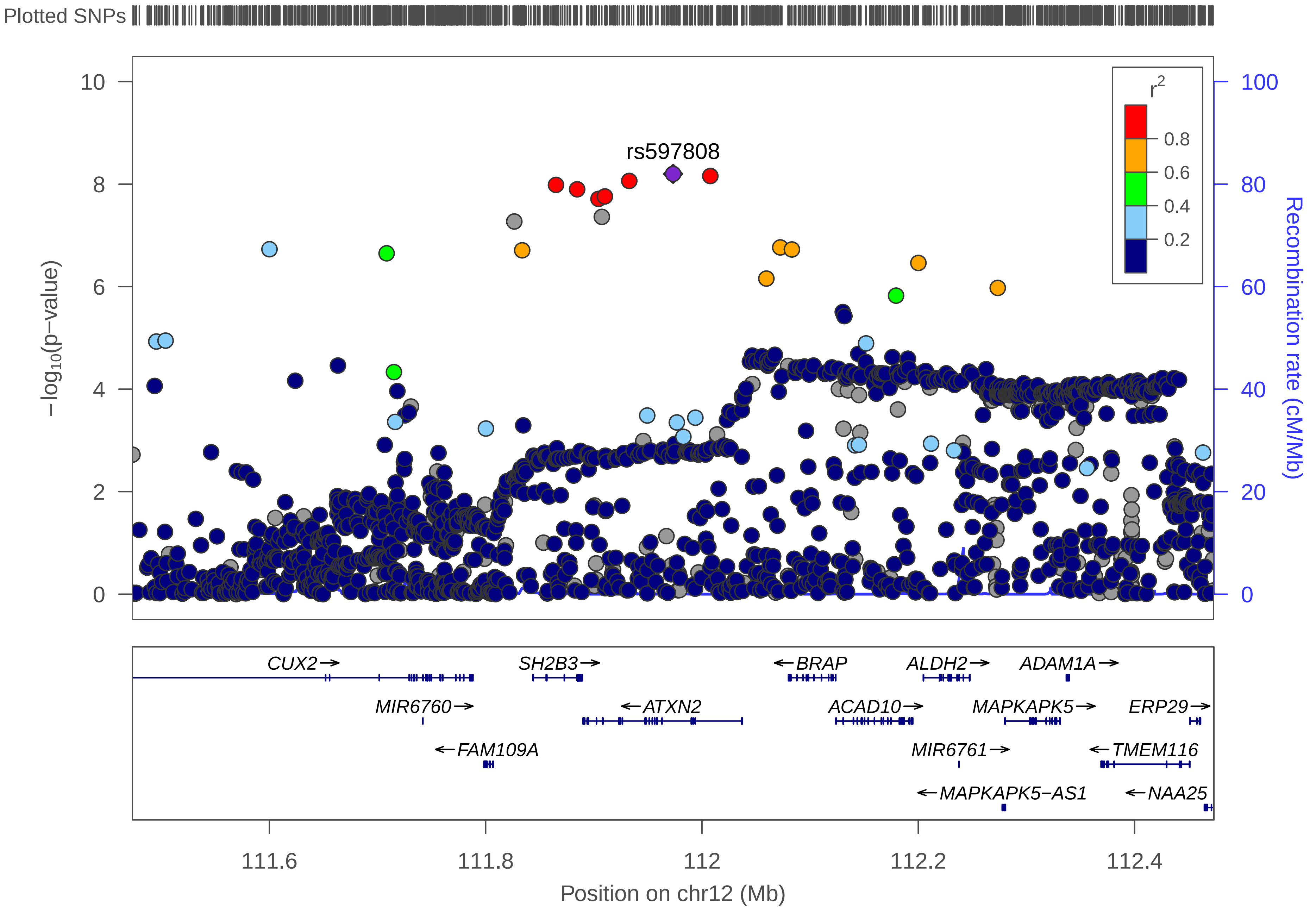

Plotted SNPs
rs597808
r²
0.8
0.6
0.4
0.2
10
8
6
4
2
0
−log10(p−value)
100
80
60
40
20
0
Recombination rate (cM/Mb)
CUX2→
SH2B3→
←BRAP
ALDH2→
ADAM1A→
MIR6760→
←ATXN2
ACAD10→
MAPKAPK5→
ERP29→
←FAM109A
MIR6761→
←TMEM116
←MAPKAPK5−AS1
←NAA25
111.6
111.8
112
112.2
112.4
Position on chr12 (Mb)

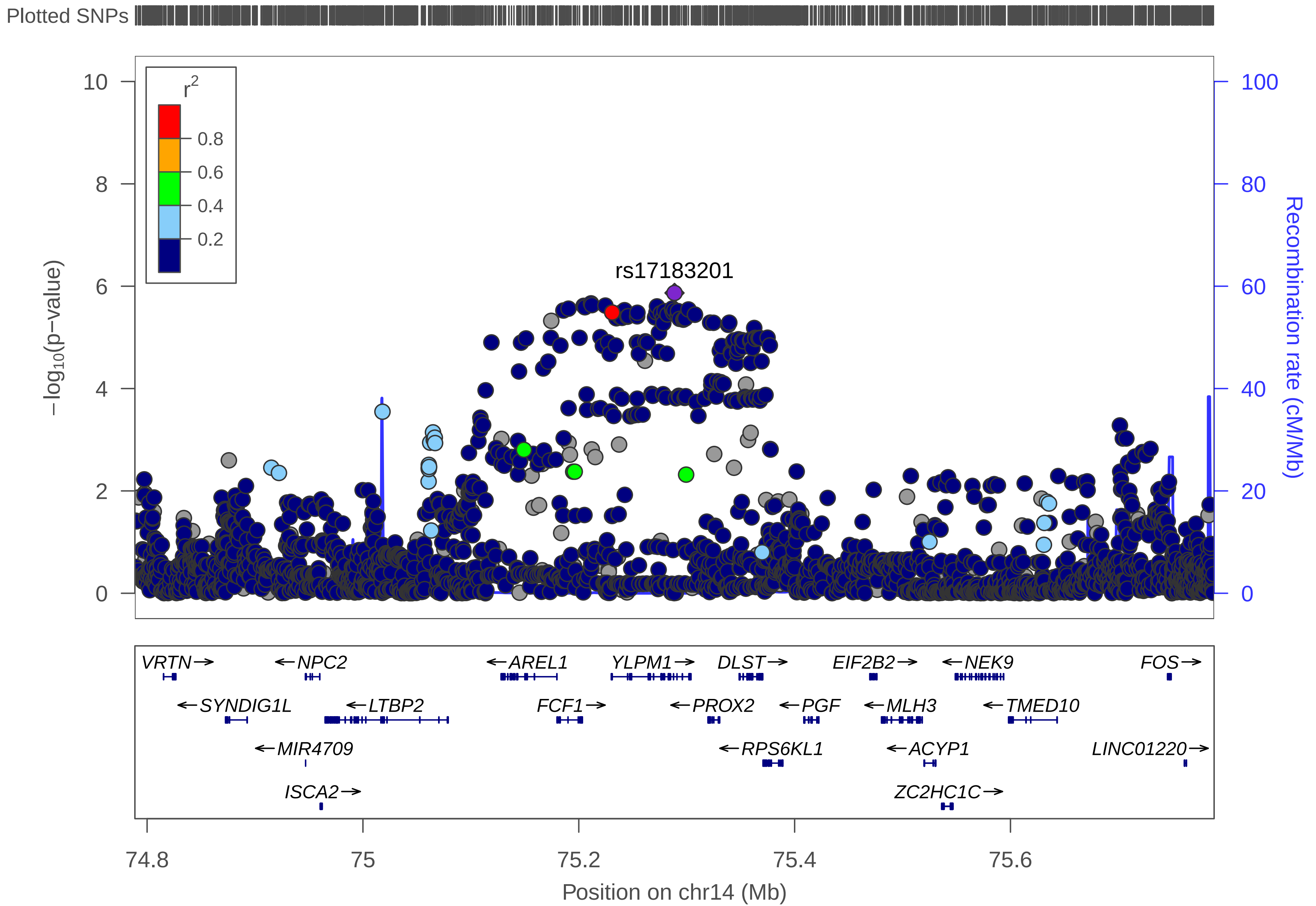
Plotted SNPs
r2
0.8
0.6
0.4
0.2
rs17183201
−log10(p−value)
Recombination rate (cM/Mb)
0
2
4
6
8
10
0
20
40
60
80
100
VRTN→
←SYNDIG1L
←MIR4709
←NPC2
ISCA2→
←LTBP2
←AREL1
FCF1→
YLPM1→
←PROX2
DLST→
←RPS6KL1
←PGF
EIF2B2→
←MLH3
←ACYP1
←NEK9
ZC2HC1C→
←TMED10
FOS→
LINC01220→
74.8
75
75.2
75.4
75.6
Position on chr14 (Mb)

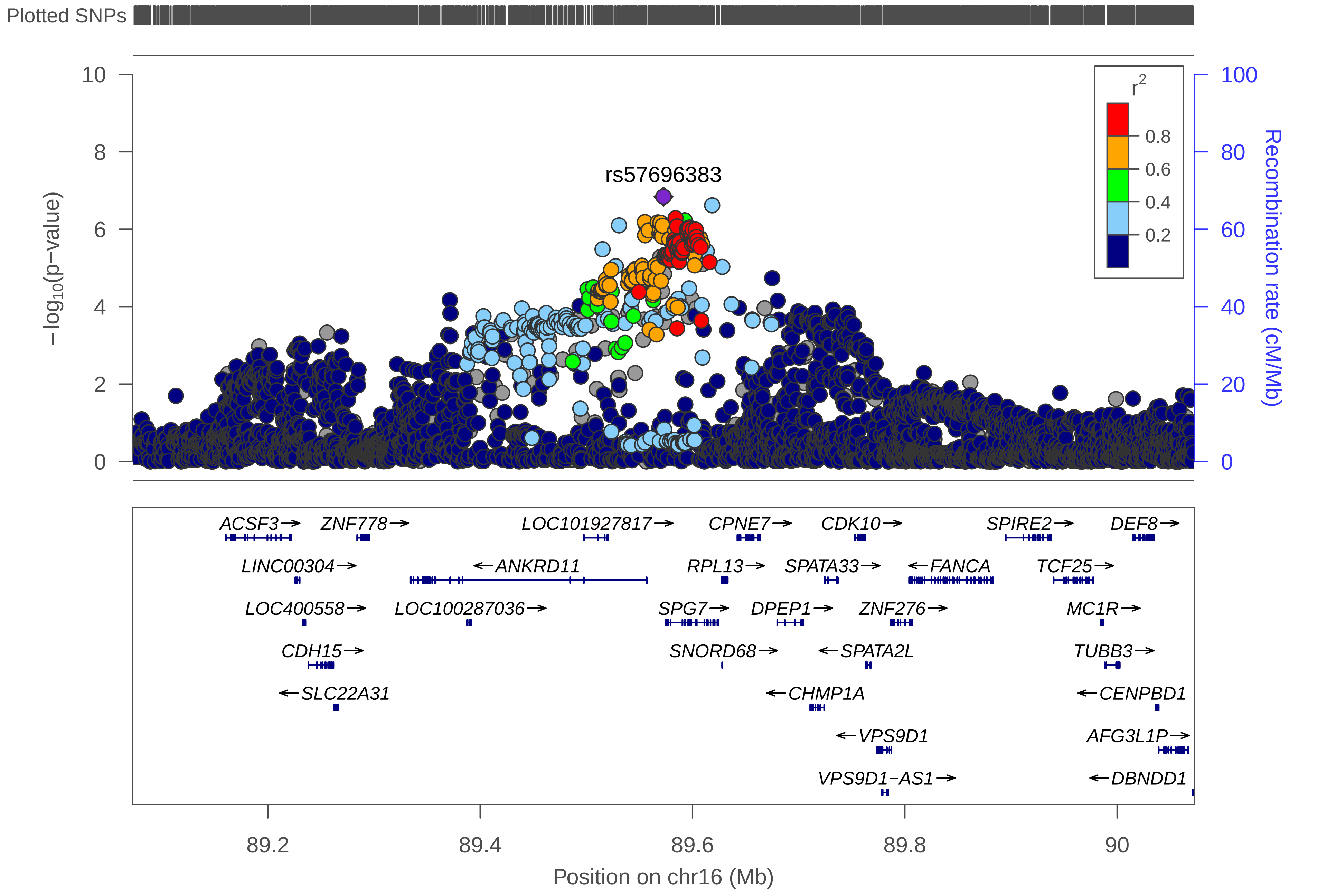
Plotted SNPs
rs57696383
r2
0.8
0.6
0.4
0.2
−log10(p−value)
Recombination rate (cM/Mb)
10
8
6
4
2
0
100
80
60
40
20
0
ACSF3→
ZNF778→
LOC101927817→
CPNE7→
CDK10→
SPIRE2→
DEF8→
LINC00304→
←ANKRD11
RPL13→
SPATA33→
←FANCA
TCF25→
LOC400558→
LOC100287036→
SPG7→
DPEP1→
ZNF276→
MC1R→
CDH15→
SNORD68→
←SPATA2L
TUBB3→
←SLC22A31
←CHMP1A
←CENPBD1
←VPS9D1
AFG3L1P→
VPS9D1−AS1→
←DBNDD1
89.2
89.4
89.6
89.8
90
Position on chr16 (Mb)

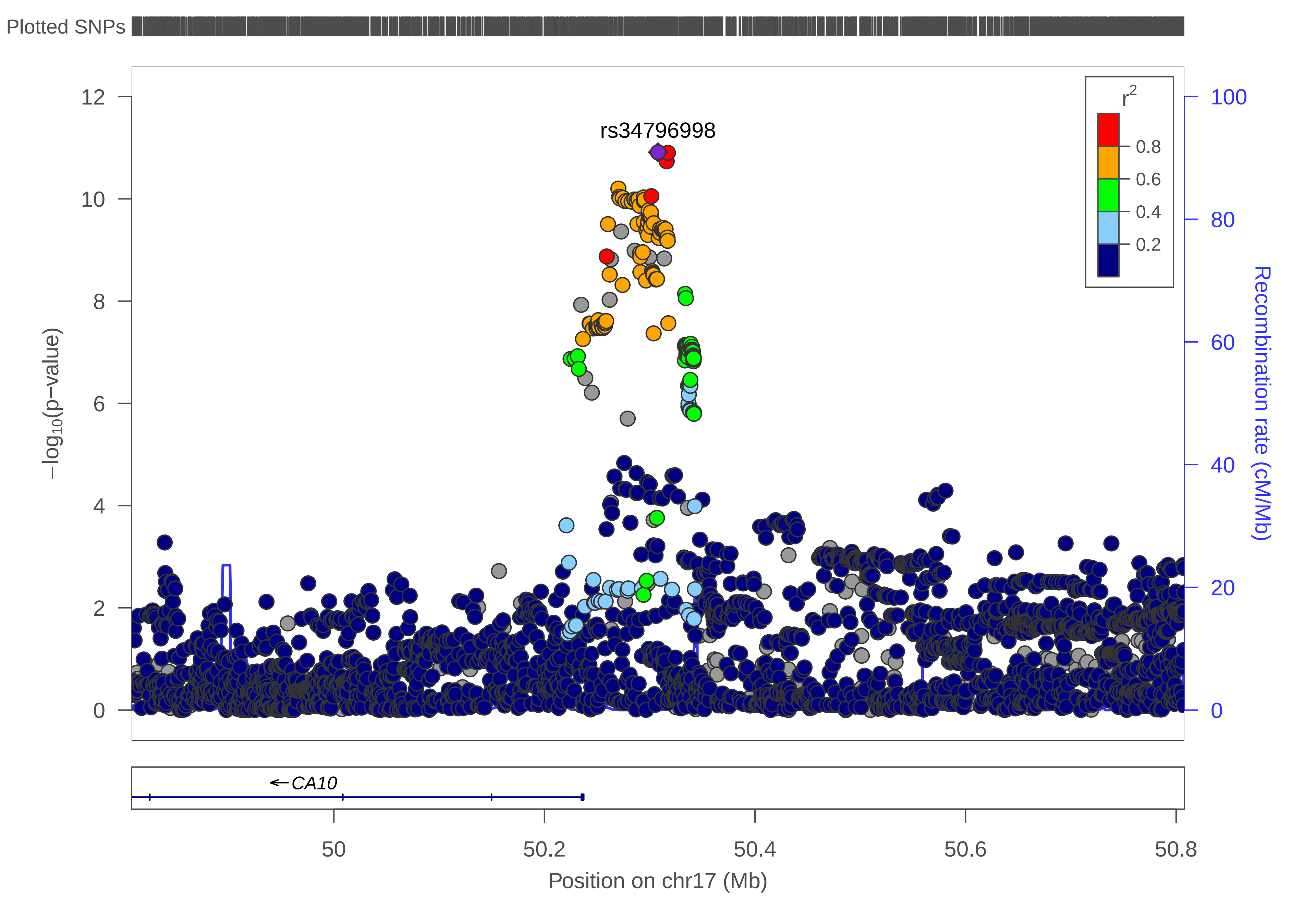

Plotted SNPs
rs34796998
r2
0.8
0.6
0.4
0.2
−log10(p−value)
0
2
4
6
8
10
12
Recombination rate (cM/Mb)
0
20
40
60
80
100
←CA10
50
50.2
50.4
50.6
50.8
Position on chr17 (Mb)

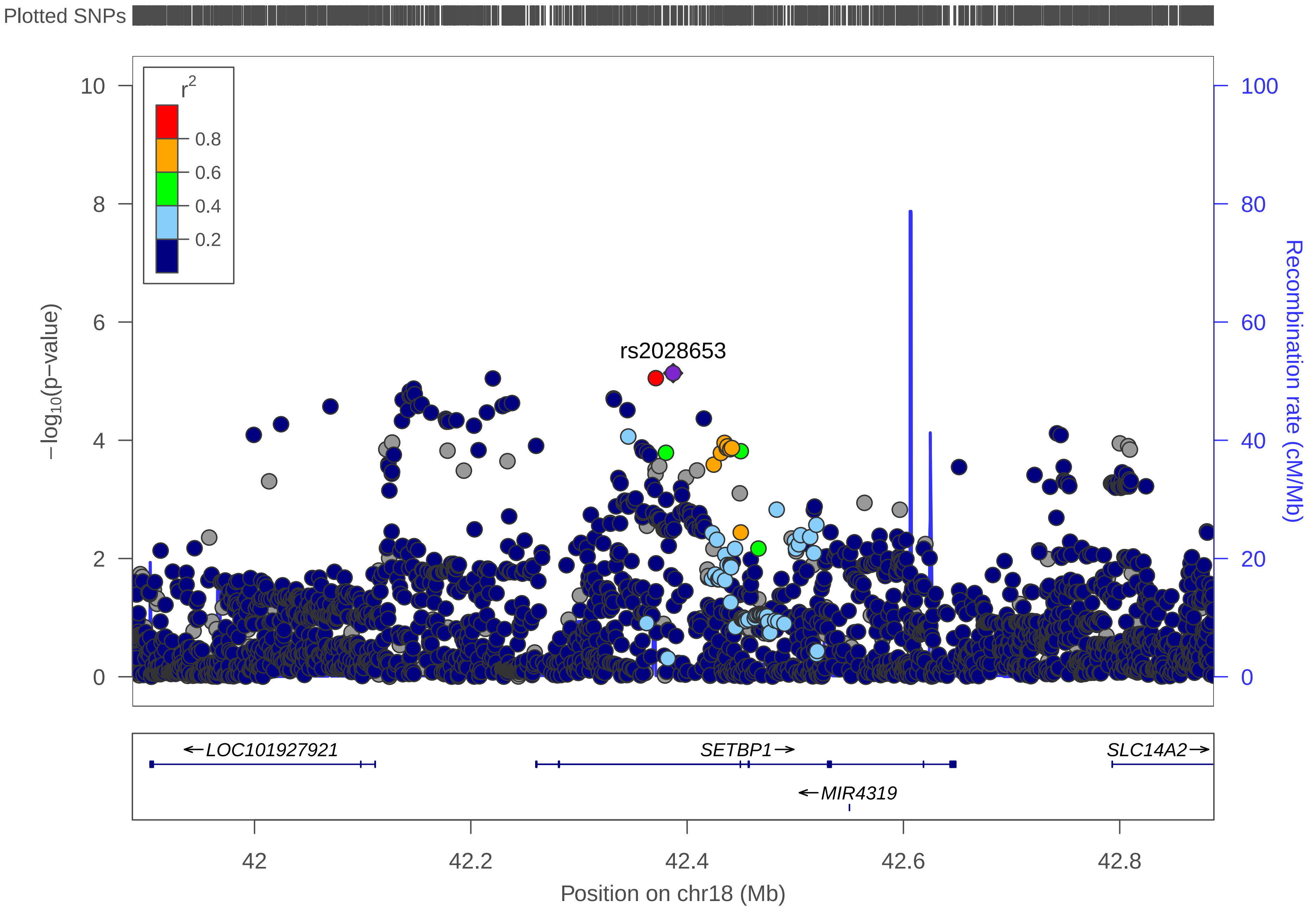

Plotted SNPs
r²
0.8
0.6
0.4
0.2
rs2028653
−log10(p−value)
Recombination rate (cM/Mb)
0
2
4
6
8
10
0
20
40
60
80
100
←LOC101927921
SETBP1→
SLC14A2→
←MIR4319
42
42.2
42.4
42.6
42.8
Position on chr18 (Mb)

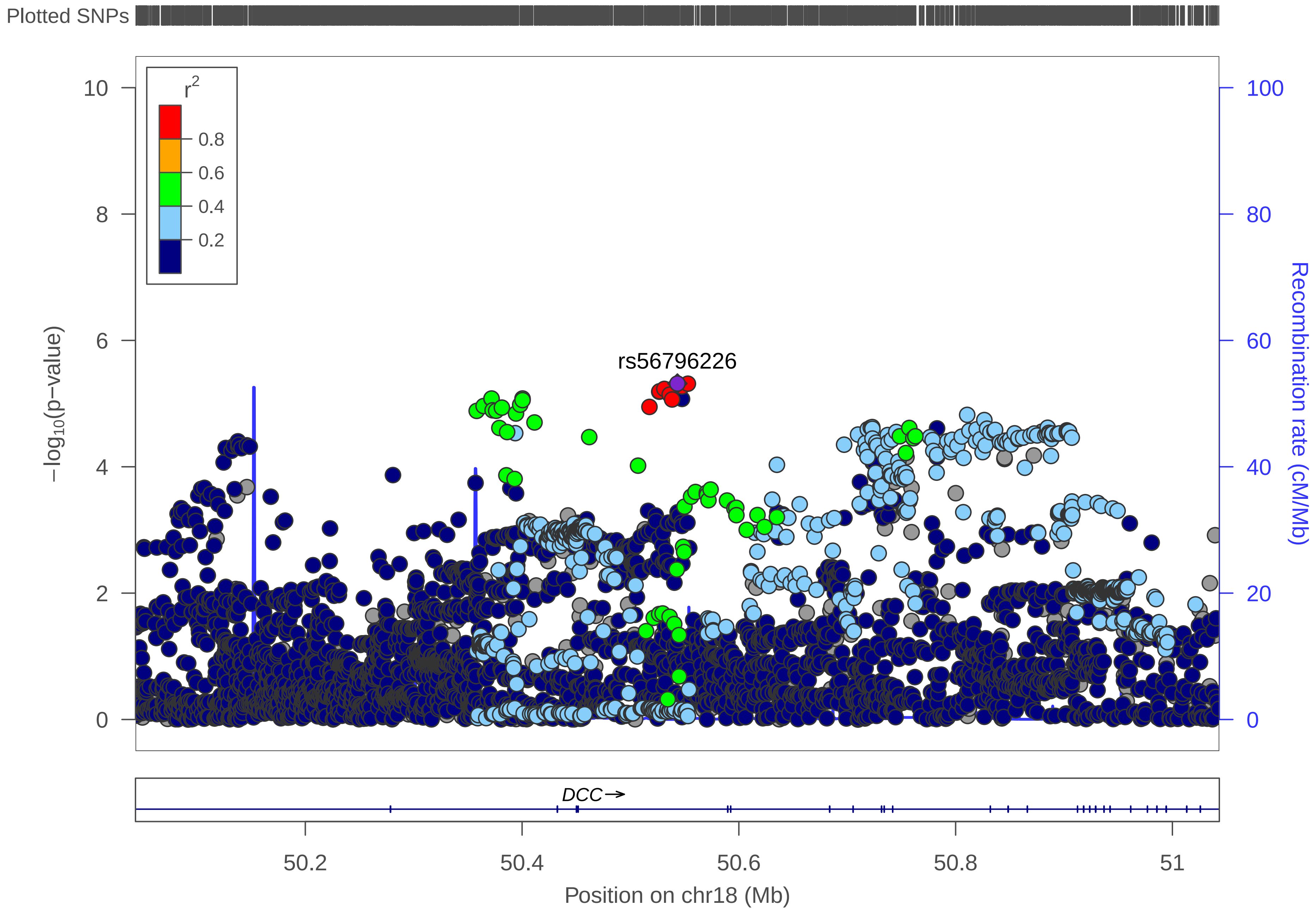
Plotted SNPs
r2
0.8
0.6
0.4
0.2
rs56796226
−log10(p−value)
Recombination rate (cM/Mb)
10
8
6
4
2
0
100
80
60
40
20
0
DCC→
50.2
50.4
50.6
50.8
51
Position on chr18 (Mb)

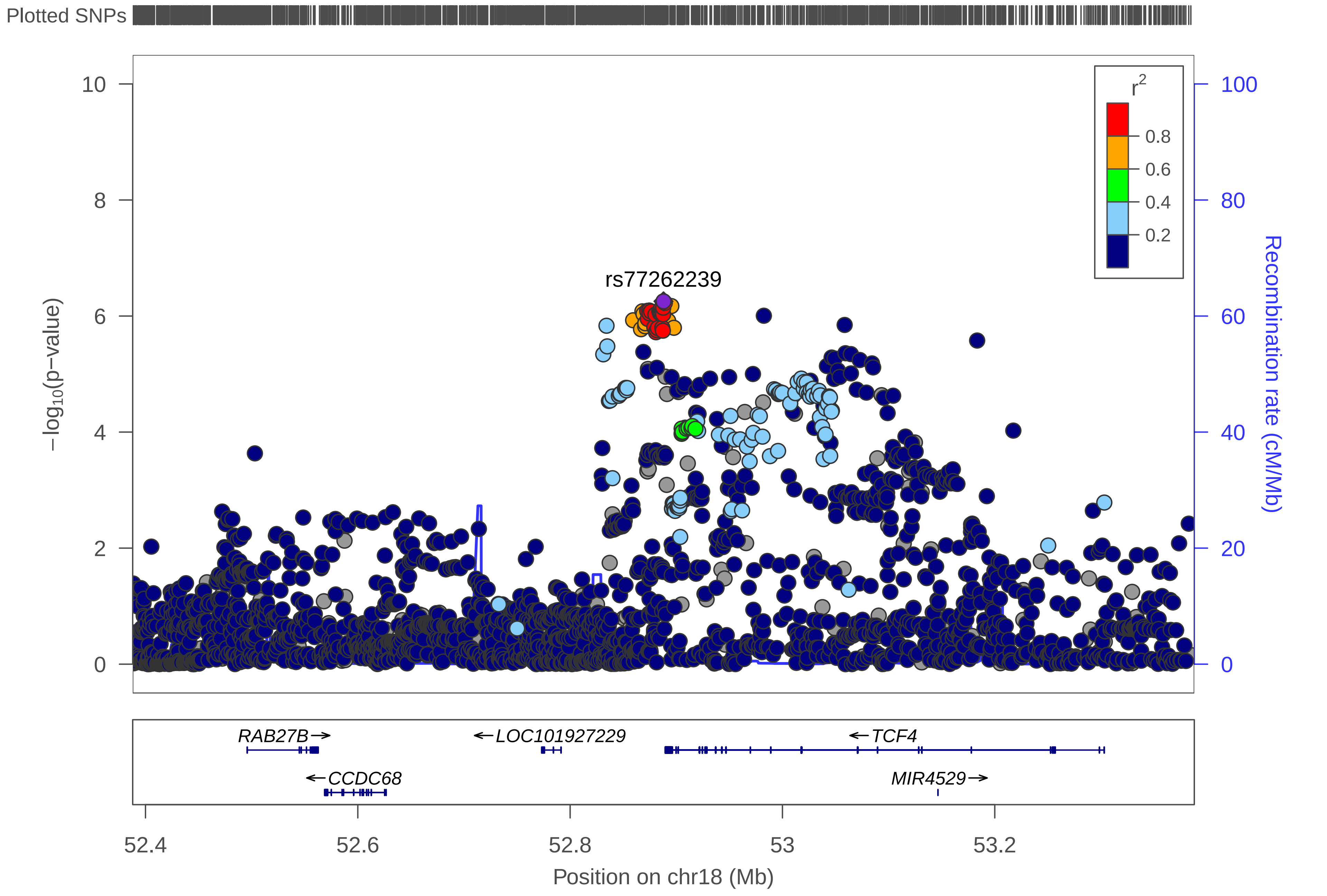

Plotted SNPs
rs77262239
r²
0.8
0.6
0.4
0.2
10
8
6
4
2
0
100
80
60
40
20
0
−log10(p−value)
Recombination rate (cM/Mb)
RAB27B→
←LOC101927229
←TCF4
←CCDC68
MIR4529→
52.4
52.6
52.8
53
53.2
Position on chr18 (Mb)

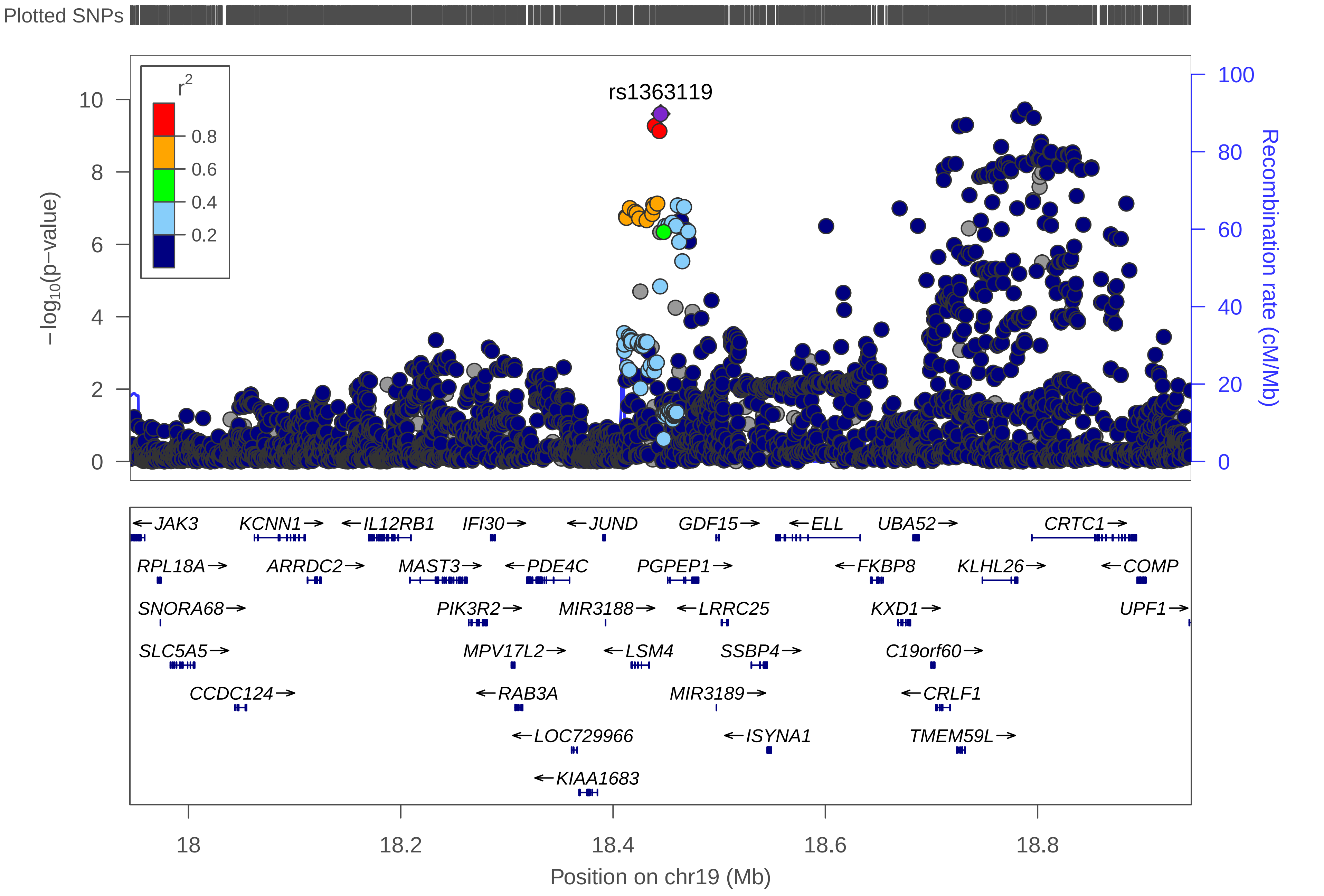
Plotted SNPs
rs1363119
r2
0.8
0.6
0.4
0.2
−log10(p−value)
Recombination rate (cM/Mb)
←JAK3
KCNN1→
←IL12RB1
IFI30→
←JUND
GDF15→
←ELL
UBA52→
CRTC1→
RPL18A→
ARRDC2→
MAST3→
←PDE4C
PGPEP1→
←FKBP8
KLHL26→
←COMP
SNORA68→
PIK3R2→
MIR3188→
←LRRC25
KXD1→
UPF1→
SLC5A5→
MPV17L2→
←LSM4
SSBP4→
C19orf60→
CCDC124→
←RAB3A
MIR3189→
←CRLF1
←LOC729966
←ISYNA1
TMEM59L→
←KIAA1683
Position on chr19 (Mb)

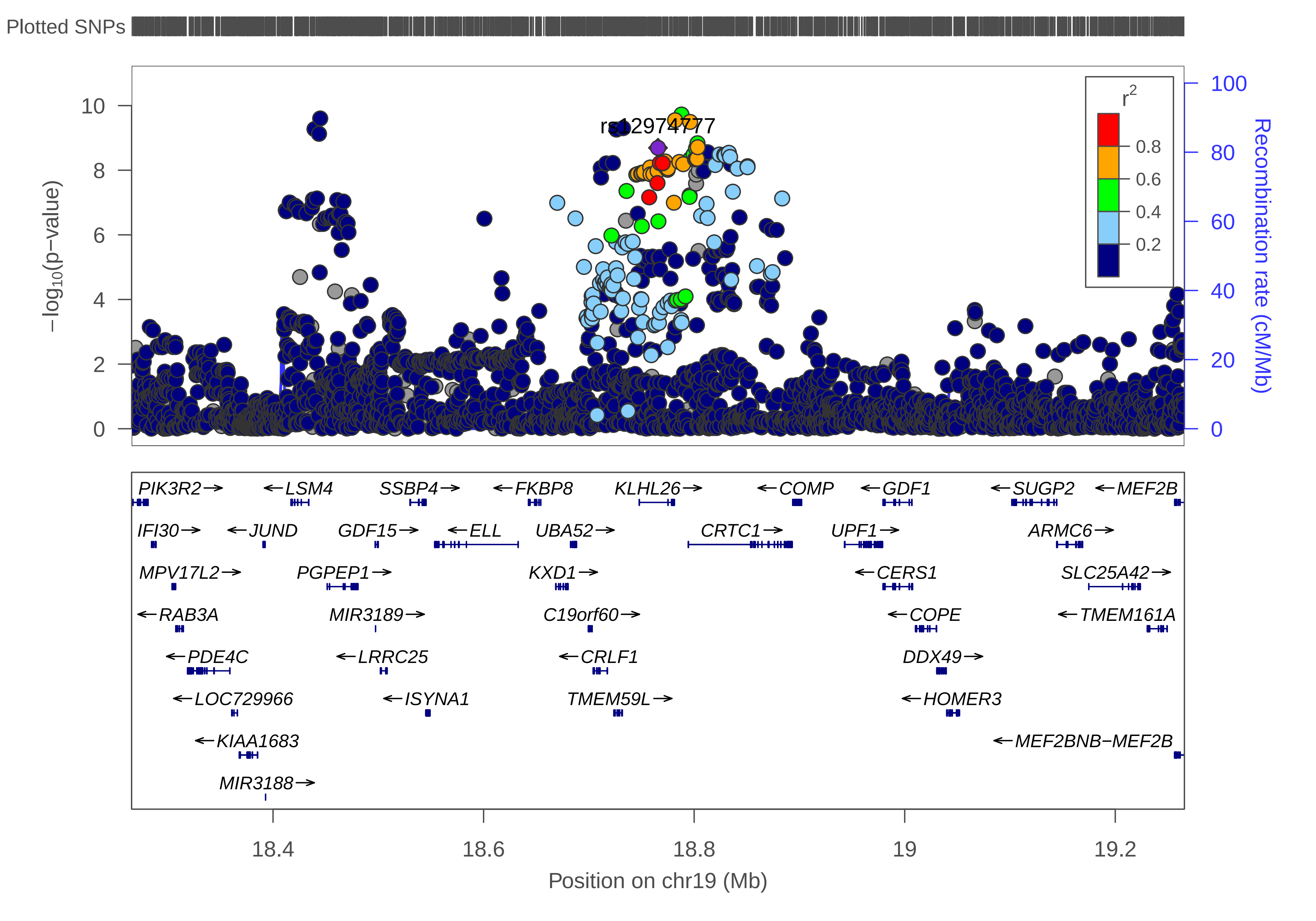

Plotted SNPs
rs12974777
r2
0.8
0.6
0.4
0.2
−log10(p−value)
Recombination rate (cM/Mb)
0
2
4
6
8
10
0
20
40
60
80
100
PIK3R2→
←LSM4
SSBP4→
←FKBP8
KLHL26→
←COMP
←GDF1
←SUGP2
←MEF2B
IFI30→
←JUND
GDF15→
←ELL
UBA52→
CRTC1→
UPF1→
ARMC6→
MPV17L2→
PGPEP1→
KXD1→
←CERS1
SLC25A42→
←RAB3A
MIR3189→
C19orf60→
←COPE
←TMEM161A
←PDE4C
←LRRC25
←CRLF1
DDX49→
←LOC729966
←ISYNA1
TMEM59L→
←HOMER3
←KIAA1683
←MEF2BNB−MEF2B
MIR3188→
18.4
18.6
18.8
19
19.2
Position on chr19 (Mb)

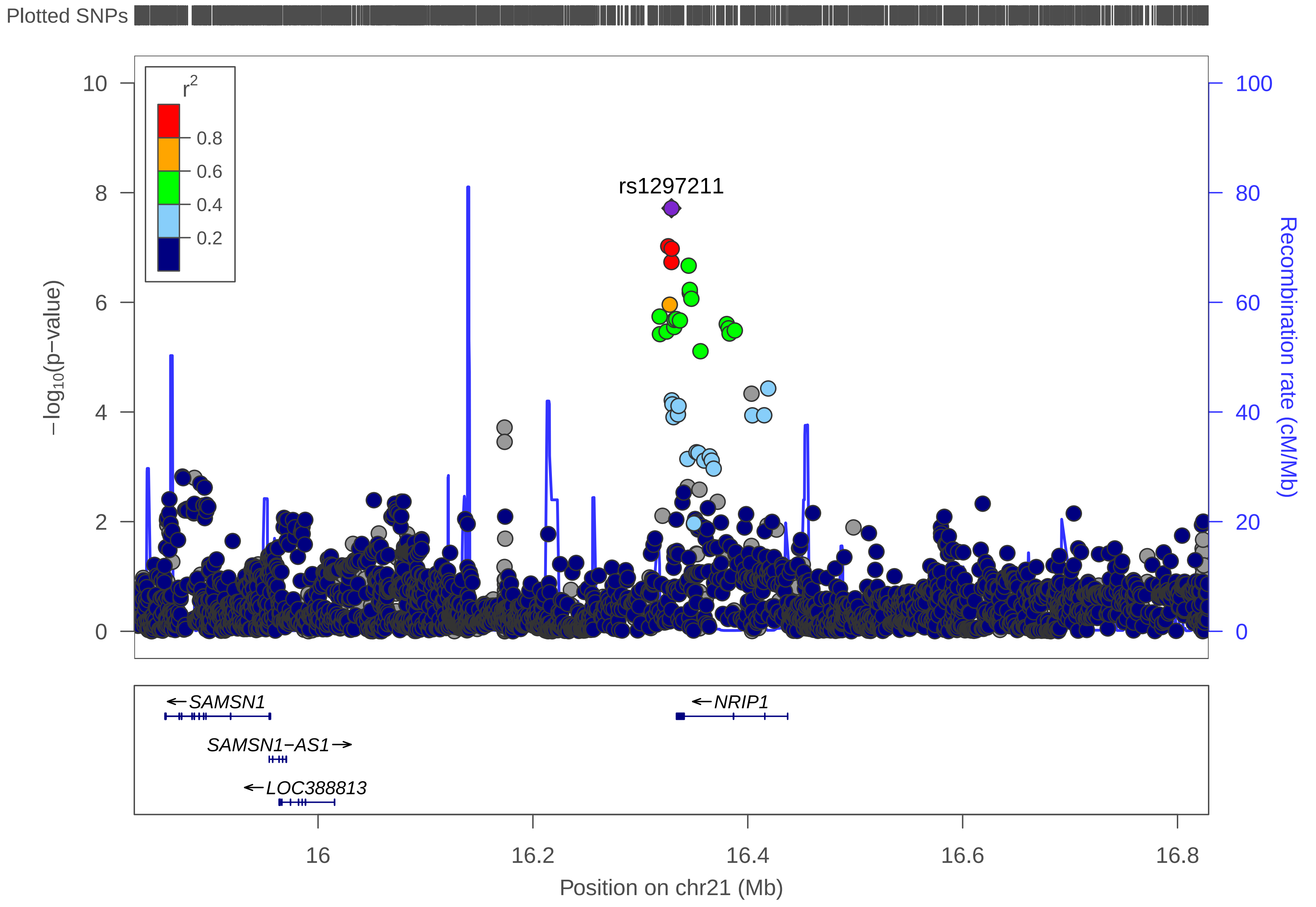

Plotted SNPs
rs1297211
r2
0.8
0.6
0.4
0.2
−log10(p−value)
10
8
6
4
2
0
Recombination rate (cM/Mb)
100
80
60
40
20
0
SAMSN1
NRIP1
SAMSN1−AS1
LOC388813
16
16.2
16.4
16.6
16.8
Position on chr21 (Mb)

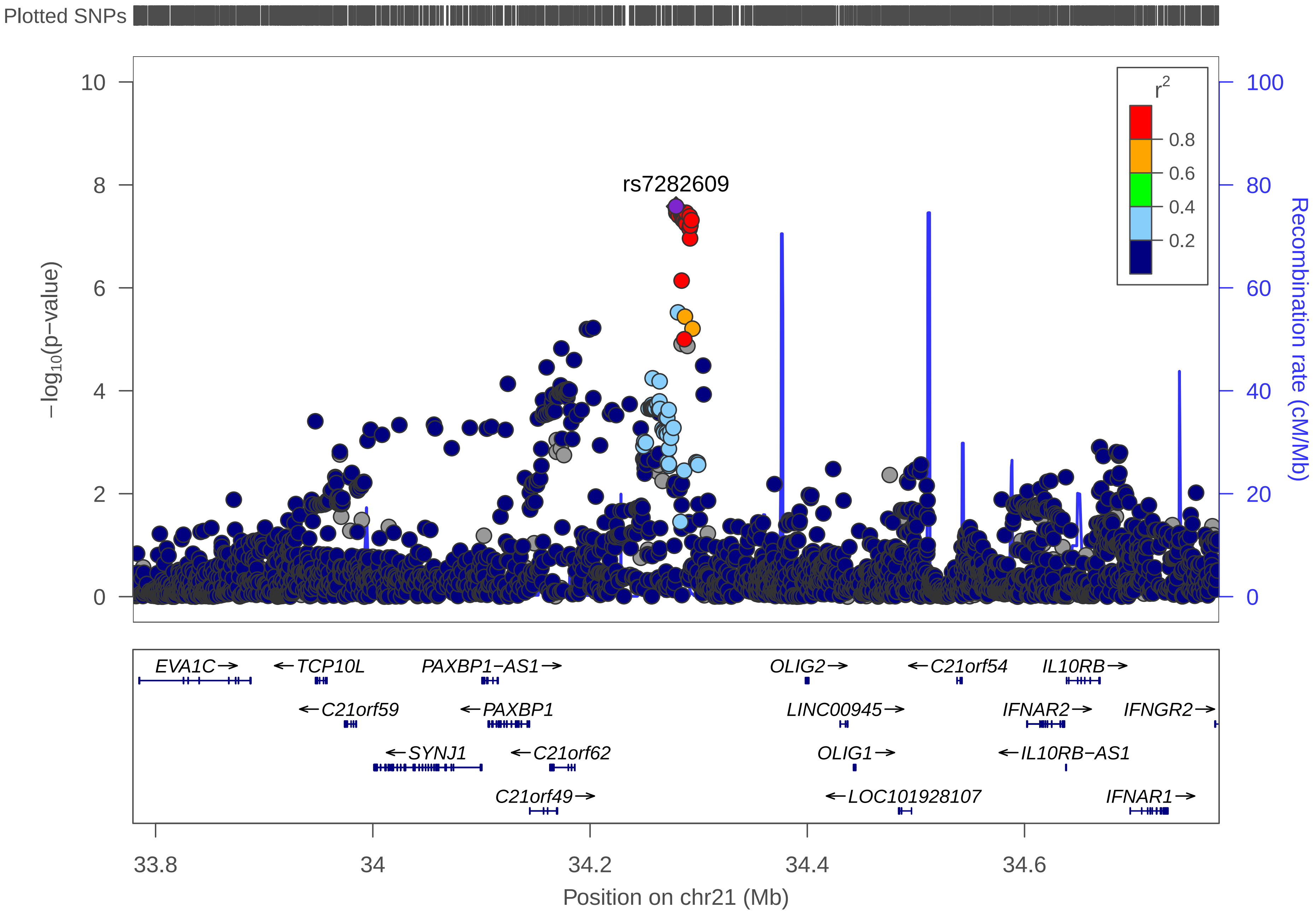

Plotted SNPs
rs7282609
−log10(p−value)
Recombination rate (cM/Mb)
r2
0.8
0.6
0.4
0.2
10
8
6
4
2
0
100
80
60
40
20
0
EVA1C→
←TCP10L
PAXBP1−AS1→
OLIG2→
←C21orf54
IL10RB→
←C21orf59
←PAXBP1
LINC00945→
IFNAR2→
IFNGR2→
←SYNJ1
←C21orf62
OLIG1→
←IL10RB−AS1
C21orf49→
←LOC101928107
IFNAR1→
33.8
34
34.2
34.4
34.6
Position on chr21 (Mb)

Supplementary Figure 3. Locuszoom plots for each genome-wide significant locus in the GERD GWAS.

Supplmentary Table 1. GERD phenotype data extraction

source	Field ID	Description	Category	code	count*
ICD10	41202	Diagnoses - main ICD10	Summary Information	K21.0/K21.9	16,420
	41204	Diagnosis - secondary ICD10	Summary Information (diagnoses)		15,419
Self-report	20002	Non-cancer illness code, self-reported	Medical conditions	1138	23,774
ICD9	41203	Diagnoses - main ICD9	Summary Information	53010/53011	17
	41205	Diagnosis - secondary ICD9	Summary Information (diagnoses)		3
OPCS	41200	Operative procedures - main OPCS	Summary Information	G24/G25	1000
	41210	Operative procedures - secondary OPCS	Summary Information (operations)		205

*count: the occurance of the corresponding code.

Supplementary Table 2. Phenotypic code exclude in control.

source	Exclude code	Data code
ICD10	K00-K93	19
Self-report	1134-1143	6
ICD9	5200-5799	87
OPCS	G-J	240

Supplementary Table 3. Extract GERD phenotype from Treatment/medication

Medications-verbal interview)	medicine	count
Reduce acid production	cimetidine	4
As above	tagamet HB	15
As above	pepcid AC	13
As above	nizatidine	103
As above	ranitidine	8,959
As above	ranitil-150mg	7
As above	Zantac	271
Block acid production and heal the esophagus	esomeprazole	1,763
As above	Nexium	882
As above	lansoprazole	19,353
As above	omeprazole	33,233
As aboves	pantoprazole	984
As above	gaviscon liquid	2,876

Antacids that neutralize stomach acid: Mylanta, Rolaids and Tums (none of them can be found in UKB)

Supplementary Table 4. Number of individuals in UKBB with GERD phenotype

phenotype	case	control	total
union (meds+self_ICD10+etc.)	75,356	279,997	355,353
union (meds+self_ICD10+etc.)	75,356	279,997	355,353
self-ICD10	43,532	279,997	323,529
ICD10-only	27,163	279,997	307,160
self_only	21,981	279,997	301,978
meds_only	58,170	279,997	338,167
CD9_only	19	279,997	280,016
operative precedure	1,101	279,997	281,098
self_ICD10	43,659	279,997	323,656

Supplmentary Table 5. BE(Barrett's esophagus) phenotype data extraction

source	Field ID	Description	Category	code	count*
ICD10	41202	Diagnoses - main ICD10	Summary Information (diagnoses)	K22.7	2,400
	41204	Diagnosis - secondary ICD10	Summary Information (diagnoses)		1,211

SuppImentary Table 6. Esophageal Adenocarcinoma phenotype data extraction

source	Field ID	Description	Category	code	count*
ICD10	40006	Type of Cancer: ICD10	Cancer register - Health-related outcomes	C15	1,536
Tumour	40011	Histology of cancer tumour	Cancer register - Health-related outcomes	8140	38360
				8141	28
				8144	271
				8147	14
				8160	301
				8190	2
				8210	306
				8211	1481
				8220	11
				8221	5
				8245	14
				8250	72
				8260	736
				8262	23
				8263	955
				8270	63
				8290	95
				8310	1301
				8313	3
				8323	32
				8330	281
				8331	17
				8332	7
				8333	2
				8400	10
				8401	38
				8408	2
				8410	18
				8480	1451
				8481	246
				8500	30746
				8503	138
				8570	21
				8571	1
				8573	6
				9014	6
				9015	4

items(8154;8161;8215;8251;8255;8322;8382;8283;8384;8413;8420;8525;8572;8576) have zero count

Supplementary Table 7. PBS code in QSKIN data

**reduce acid production:**	
Code & Prescriber	medical product
1158Y	cimetidine
2487X,2488Y	pepcid AC
1504E,1505F	nizatidine
1977C,1978D,1937Y,8162N	ranitidine
**block acid production**	
Code & Prescriber	medical product
3401B,8601Q,10295W,10343J,8600P,8886Q,10330Q,10331R	esomeprazole
9477T,9478W,8198L,2240X,2241Y,9331D	lansoprazole
1326T,1327W,8332M,8331L,8333N,9109K,9110L	omeprazole
8399C,9423Y,9424B,8007K,8008L	pantoprazole
8507R, 8508T, 8509W	RABEPRAZOLE

Supplmentary Table 8. Number of controls in BE and EA cohorts

	UKBB	BEACON	Cambridge	Oxford	Bonn	total
BE	2,831	2,406	873	1,851	1,037	8,998
EA	568	1,508	995	N/A	1,609	4,680
#control	2,088	6,718	3,408	3,496	3,537	19,247

Supplmentary Table 9.Top SNPs in BEEA in GERD GWAS after excluding BE/EA cases

SNP	Chr	Position	Tested allele	Other allele	Nearest gene or region	effect	se	pvalue
rs7255	2	20878820	T	C	GDF7 and LDAH	0.0236	0.0056	2.73E-05
rs13960654	2	200045039	T	C	SATB2	0.0154	0.0059	9.71E-03
rs2687202	3	70929983	T	C	FOXP1	0.0341	0.0061	2.61E-08
rs9918259(	5	663092	T	C	TPPP and CEP72	-0.0311	0.007	9.80E-06
rs9257809	6	29356331	A	G	MHC region	0.0464	0.0085	5.00E-08
rs62423175	6	62195368	A	G	KHDRBS2 and MTRNR2L9	-0.0071	0.0076	3.50E-01
rs17451754	7	117256712	A	G	CFTR	0.0129	0.0082	1.17E-01
rs17749155	8	10068073	A	G	MSRA	-0.0023	0.0076	7.67E-01
rs10108511	8	11435516	T	C	LINC00208and BLK	0.0053	0.0056	3.42E-01
rs7852462	9	100310501	T	C	TMOD1	0.0066	0.0058	2.54E-01
rs1247942	12	114673723	C	G	LOC105369996and TBX5	0.0149	0.0057	8.75E-03
rs2464469	15	58362025	A	G	ALDH1A2	0.0034	0.0057	5.47E-01
rs1979654	16	86396835	C	G	LOC732275and FOXF1	0.0001	0.0057	9.98E-01
rs19962055	19	18804294	T	TG	CRTC1	0.0254	0.0063	5.19E-05
