# Supplementary material for: Gastroesophageal reflux GWAS identifies risk loci that also associate with subsequent severe esophageal diseases
Source: Nat Commun. 2019 Sep 16;10:4219. doi: 10.1038/s41467-019-11968-2 (PMC6746768; doi:10.1038/s41467-019-11968-2)
Supplement: Supplementary file 3 — Reporting Summary [file 41467_2019_11968_MOESM3_ESM.pdf]

nature research

Corresponding author(s): Stuart Macgregor

Last updated by author(s): Dec 22, 2018

Reporting Summary

Nature Research wishes to improve the reproducibility of the work that we publish. This form provides structure for consistency and transparency in reporting. For further information on Nature Research policies, see Authors & Referees and the Editorial Policy Checklist.

Statistics

For all statistical analyses, confirm that the following items are present in the figure legend, table legend, main text, or Methods section.

n/a	Confirmed	
☐	☒	The exact sample size (n) for each experimental group/condition, given as a discrete number and unit of measurement
☐	☒	A statement on whether measurements were taken from distinct samples or whether the same sample was measured repeatedly
☐	☒	The statistical test(s) used AND whether they are one- or two-sided<br>*Only common tests should be described solely by name; describe more complex techniques in the Methods section.*
☐	☒	A description of all covariates tested
☐	☒	A description of any assumptions or corrections, such as tests of normality and adjustment for multiple comparisons
☐	☒	A full description of the statistical parameters including central tendency (e.g. means) or other basic estimates (e.g. regression coefficient) AND variation (e.g. standard deviation) or associated estimates of uncertainty (e.g. confidence intervals)
☐	☒	For null hypothesis testing, the test statistic (e.g. F, t, r) with confidence intervals, effect sizes, degrees of freedom and P value noted<br>*Give P values as exact values whenever suitable.*
☒	☐	For Bayesian analysis, information on the choice of priors and Markov chain Monte Carlo settings
☒	☐	For hierarchical and complex designs, identification of the appropriate level for tests and full reporting of outcomes
☐	☒	Estimates of effect sizes (e.g. Cohen's d, Pearson's r), indicating how they were calculated

Our web collection on statistics for biologists contains articles on many of the points above.

Software and code

Policy information about availability of computer code

Data collection	na
Data analysis	For gene-based and gene-set analyses we used MAGMA 1.06<br>SNP heritability and genetic correlations were estimated using LD score regression (https://github.com/bulik/ldsc) and LD hub (http://ldsc.broadinstitute.org/).<br>eQTL for 44 GTEx tissues (https://gtexportal.org/)<br>PheWAS and drug target information (https://genetics.opentargets.org)

For manuscripts utilizing custom algorithms or software that are central to the research but not yet described in published literature, software must be made available to editors/reviewers. We strongly encourage code deposition in a community repository (e.g. GitHub). See the Nature Research guidelines for submitting code & software for further information.

Data

Policy information about availability of data

All manuscripts must include a data availability statement. This statement should provide the following information, where applicable:

- Accession codes, unique identifiers, or web links for publicly available datasets
- A list of figures that have associated raw data
- A description of any restrictions on data availability

Genotype and phenotype data from UKBB have already been processed and are available at https://www.ukbiobank.ac.uk/. The GWAS result from 23andMe are available by request from https://www.23andme.com/. Data from QSKIN are available on request from project leader Professor David Whiteman david.whiteman@qimrberghofer.edu.au.

Field-specific reporting

Please select the one below that is the best fit for your research. If you are not sure, read the appropriate sections before making your selection.

☒ Life sciences ☐ Behavioural & social sciences ☐ Ecological, evolutionary & environmental sciences

For a reference copy of the document with all sections, see nature.com/documents/nr-reporting-summary-flat.pdf

Life sciences study design

All studies must disclose on these points even when the disclosure is negative.

Sample size	Rather than generate a sample size calculation we assembled the largest possible GWAS dataset to identify novel loci. This approach was successful as we identified numerous novel loci.
Data exclusions	Samples excluded during GWAS quality control, including for ancestry, data quality and relatedness, are described in full in the methods section.
Replication	Data was collected from multiple independent cohorts.
Randomization	Samples were derived from existing cohorts and were not randomized.
Blinding	GWAS cleaning and quality control was performed blind to the phenotypes.

Reporting for specific materials, systems and methods

We require information from authors about some types of materials, experimental systems and methods used in many studies. Here, indicate whether each material, system or method listed is relevant to your study. If you are not sure if a list item applies to your research, read the appropriate section before selecting a response.

Materials & experimental systems

n/a	Involved in the study	
☒	☐	Antibodies
☒	☐	Eukaryotic cell lines
☒	☐	Palaeontology
☒	☐	Animals and other organisms
☐	☒	Human research participants
☒	☐	Clinical data

Methods

n/a	Involved in the study	
☒	☐	ChIP-seq
☒	☐	Flow cytometry
☒	☐	MRI-based neuroimaging

Human research participants

Policy information about studies involving human research participants

Population characteristics	Described in full in the methods section.
Recruitment	Described in full in the methods section.
Ethics oversight	Described in full in the methods section.

Note that full information on the approval of the study protocol must also be provided in the manuscript.
