# Supplementary material for: Gastroesophageal reflux GWAS identifies risk loci that also associate with subsequent severe esophageal diseases
Source: Nat Commun. 2019 Sep 16;10:4219. doi: 10.1038/s41467-019-11968-2 (PMC6746768; doi:10.1038/s41467-019-11968-2)
Supplement: Supplementary file 4 — Description of Additional Supplementary Files [file 41467_2019_11968_MOESM4_ESM.pdf]

Title: Supplementary Data 1.
Description: 25 significantly associated genomic regions in meta-analysis of GERD

Title: Supplementary Data 2.
Description: LD hub results

Title: Supplementary Data 3.
Description: Gene ATLAS result

Title: Supplementary Data 4.
Description: Drugs in clinical trials

Title: Supplementary Data 5.
Description: MAGAMA gene based test results

Title: Supplementary Data 6.
Description: MetaXcan gene based test results

Title: Supplementary Data 7.
Description: Gene set enrichment analysis using DEPICT

Title: Supplementary Data 8.
Description: GERD GWAS after excluding BE/EA cases
